# Analysis of Volatile Components of Jasmine and Jasmine Tea during Scenting Process

**DOI:** 10.3390/molecules27020479

**Published:** 2022-01-12

**Authors:** Yangbo Zhang, Yifan Xiong, Huimin An, Juan Li, Qin Li, Jianan Huang, Zhonghua Liu

**Affiliations:** 1 Key Laboratory of Tea Science of Ministry of Education, Hunan Agricultural University, Changsha 410128, China; zhangyblucky@163.com (Y.Z.); xyf951118@163.com (Y.X.); anhuimin1995@126.com (H.A.); xixi_lj@126.com (J.L.); liqinvip@126.com (Q.L.); 2 National Research Center of Engineering and Technology for Utilization of Botanical Functional Ingredients, Hunan Agricultural University, Changsha 410128, China; 3 Co-Innovation Center of Education Ministry for Utilization of Botanical Functional Ingredients, Hunan Agricultural University, Changsha 410128, China

**Keywords:** volatile components, jasmine tea, traditional scenting process, new scenting process, JTF index

## Abstract

Jasmine tea is widely loved by the public because of its unique and pleasant aroma and taste. The new scenting process is different from the traditional scenting process, because the new scenting process has a thin pile height to reduce the high temperature and prolong the scenting time. We qualified and quantified volatiles in jasmine and jasmine tea during the scenting process by gas chromatography-mass spectrometry (GC-MS) with a headspace solid-phase microextraction (HS-SPME). There were 71 and 78 effective volatiles in jasmine and jasmine tea, respectively, including 24 terpenes, 9 alcohols, 24 esters, 6 hydrocarbons, 1 ketone, 3 aldehydes, 2 nitrogen compounds, and 2 oxygen-containing compounds in jasmine; 29 terpenes, 6 alcohols, 28 esters, 8 nitrogen compounds, 1 aldehyde, and 6 other compounds in jasmine tea. The amounts of terpenes, esters, alcohols, nitrogen compounds, and hydrocarbons in jasmine and tea rose and then fell. The amount of oxygenated compounds of tea in the new scenting process first rose and then fell, while it showed a continuous upward trend during the traditional process. The amount of volatiles in jasmine and tea produced by the new scenting process were higher than that of the traditional scenting process at the same time. This study indicated that jasmine tea produced by the new scenting process had better volatile quality, which can provide proof for the new scenting process.

## 1. Introduction

Jasmine tea is deeply loved by consumers in the northern and southeastern areas of China due to its pleasant aroma and health benefits [1,2,3,4]. Traditional jasmine tea is made by repeatedly mixing tea dhool with *Jasminum sambac* to ensure a strong fragrance, including preprocessing tea dhool, maintaining fresh jasmine, mixing jasmine and tea dhool, spreading out the mixture for heat dissipation, reheating and scenting, separating jasmine from the tea, drying, and cooling [4]. 

Aroma is an important component in jasmine and jasmine tea. Chen Q et al. [5] analyzed the volatile substances released by jasmine in a natural state and found that those components were highly consistent with the aroma smelled in the sensory review. Another study found that the fragrance of jasmine had a potential correlation with the peak area percentages of α-farnesene, (Z)-3-hexenyl benzoate, 2-methyl anthranilate, indole, and linalool, which is called the “jasmine tea flavor index” (JTF) [6]. The main volatile compounds in jasmine were α-farnesene, (Z)-3-hexenyl benzoate, linalool, benzyl alcohol, benzyl acetate, methyl anthranilate, and indole [7,8,9,10]. Previous studies have detected the main aroma components in jasmine tea, including cis-3-hexene benzoate, benzyl acetate, methyl 2-aminobenzoate, linalool, (Z)-3-hexanol, methyl salicylate, and benzyl alcohol [8,9,11,12]. It has been proven that the intensity of sweetness and substances of jasmine tea were decreased by 4-caprolactone [13]. Other studies also showed that cis-3-hexenyl acetate, linalool, methyl benzoate, benzyl acetate, α-farnesene, methyl salicylate, methanol benzene, benzoic acid-cis-3-hexenyl ester, methyl anthranilate, and indole were the main volatile components in jasmine tea [14,15], which mainly exist in the body of jasmine as bonded glycosides and are released through endogenous sources. It has also been reported that aromas in jasmine are released by enzymatic hydrolysis, and β-primrose glycosidase and β-glucosidase played a key role during this process [16]. Li H et al. [9] analyzed the influence of different processes on the aroma quality of jasmine tea and found that there were 130 aroma components and 20 characteristics in the traditional scenting process (TSP). 

The quality of jasmine tea could be affected by the quality of jasmine, tea dhools, scenting technique (methods and technological parameters), and so on [5,9]. In the conventional view, the quality of jasmine tea has a positive correlation with the rounds of the scenting process [17]. However, the profit will be limited because of the higher cost, lower efficiency, and longer and more complicated scenting process [5]. To avoid the shortcomings in the traditional scenting process, our previous studies proposed a new jasmine tea scenting process to avoid the abovementioned problems [18,19]. The objective of this study is to investigate the changes in aroma components and characteristics of jasmine and jasmine tea in different scenting processes during different times and to study the differences in aromas in jasmine tea made by new scenting processes and traditional scenting processes. To reach this goal, we used HS-SPME and GC-MS combined with qualitative and quantitative analysis and combined principal component analysis (PCA) and multivariate statistical analysis methods to analyze the volatiles in jasmine and jasmine tea during the different scenting processes, which could provide a new view of the scenting process of jasmine tea. 

## 2. Results

### 2.1. Changes in Water, Temperature, and Humidity during Scenting Processes

**Figure 1:**
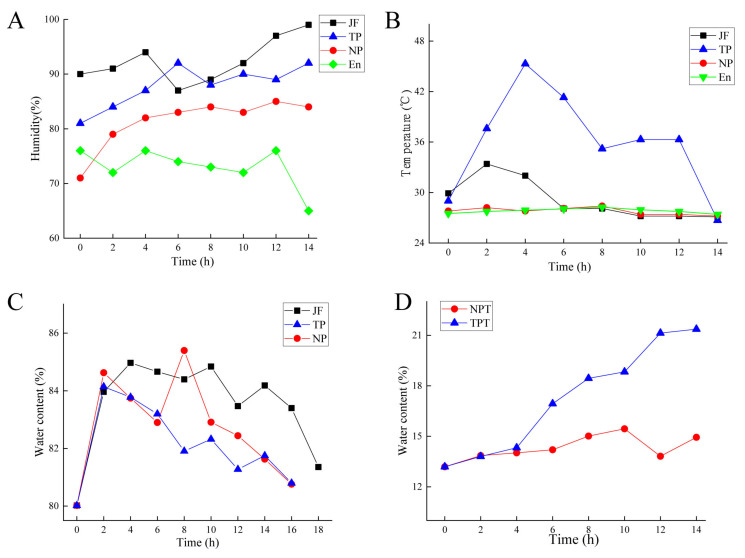
The change during scenting process. (**A**) humidity change of jasmine. (**B**) temperature change of jasmine. (**C**) water content of scented jasmine. (**D**) water content of jasmine tea. JF: jasmine flower; NP: jasmine flower in the new scenting process; TP: jasmine flower in the traditional scenting process; NPT: jasmine tea in the new scenting process; TPT: jasmine tea in the traditional scenting process; En: environment.

The changes in humidity and temperature during the scenting process are shown in Figure 1A,B. The humidity presented an upward trend. The temperatures of TP (jasmine in the traditional scenting process) and JF (isolated jasmine) showed a trend of rising and falling. After 4 h of scenting, the pile temperature of the traditional scenting process reached the highest point, which indicated that the samples needed to be turned over. After the pile was turned over, the temperature showed a downward trend. The water content of isolated jasmine, jasmine, and tea at different scenting processes during different times was measured. The results are shown in Figure 1C,D. The water contents of jasmine all showed a trend of first increasing and then decreasing. Among the jasmine teas, the NPT (jasmine tea in the new scenting process) showed an upward trend but remained relatively lower than TPT (jasmine tea in the traditional scenting process). There was a continuous upward trend in TPT, and the highest water content was 22%.

### 2.2. Qualitative Analysis of Volatile Components in Jasmine and Jasmine Tea

#### 2.2.1. Qualitative Analysis of Volatile Components in Jasmine 

The volatile components of jasmine are shown in Supplementary Materials Table S2. There were 9 alcohols, 24 esters, 24 terpenes, 6 hydrocarbons, 1 ketone compound, 3 aldehydes, 2 nitrogen compounds, and 2 oxygens for a total of 71 kinds of volatile aroma component.

#### 2.2.2. Qualitative Analysis of Volatile Components in Jasmine Tea 

The qualitative analysis results of the volatile components of the tea are shown in Supplementary Table S4. There were 78 kinds of volatile compound in jasmine tea, including 6 alcohols, 28 esters, 29 terpenes, 8 nitrogen-containing compounds, 1 aldehyde compound, and 6 other compounds.

#### 2.2.3. Fragrance Evaluation Index (JTF)

The JTF index was directly proportional to aroma concentration and freshness. Therefore, the quality of the aroma was evaluated by JTF.

JTF index = (α-Farnesene + 3-Hexenol Benzoate + Indole + 2-Methyl Aminobenzoate)/Linalool(1)

**Figure 2:**
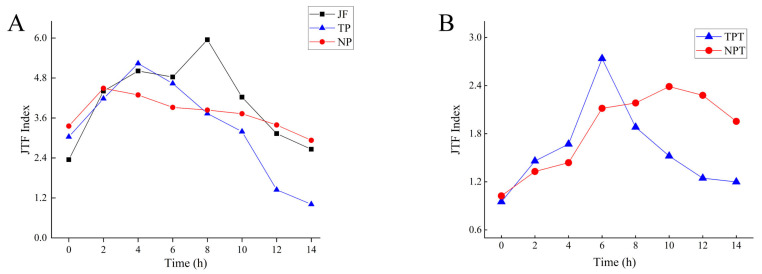
Changes in JTF index. (**A**) JTF of jasmine. (**B**) JTF of jasmine tea.

During the scenting process, the JTF indices of jasmine all showed a trend of increasing and then decreasing (Figure 2A). Compared with TP, the change in the JTF index of NP (jasmine in the new scenting process) was relatively small and maintained at a high level, which was similar to the changing trend of the JTF index of JF (Figure 2A). The aroma evaluation index of jasmine tea also showed a trend of increasing and decreasing but was slightly different from that of jasmine (Figure 2B). 

### 2.3. Quantitative Analysis of Volatile Components 

#### 2.3.1. Quantitative Analysis of Volatile Components in Jasmine 

##### Establishment of Standard Curve of Jasmine 

During the scenting process, jasmine was collected every two hours during the whole experiment to serve as the QC (quality control) samples. A quantity of 1000 g jasmine was weighed for the QC samples, in which the jasmine samples were taken without any treatment. QC samples from all samples were used to prepare blank substrates for jasmine, which could prevent the matrix effect from interfering with the volatile components of jasmine. Twenty-four standard curves for the volatile components of jasmine were established (as shown in Supplementary Table S1) for quantitative analysis after optimizing the extraction conditions and GC-MS conditions. The R^2^ values of the standard curves of the 24 chemical components were all between 0.9000 and 0.9999, which showed that the standard curve established in this experiment could more accurately quantitatively analyze the volatile components to be tested. Among them, benzyl acetate had a low response value in GC-MS; its linear range was 1.996~79,920.0000 μg·L^−1^, and its detection limit and quantification limit were also the highest, 0.3842 μg·L^−1^ and 1.1672 μg·L^−1^, respectively. The linear range of limonene was the smallest, from 2.013 to 50.325 μg·L^−1^; the lowest detection limit was in limonene and folate 2-methyl butyrate, both 0.0010 μg·L^−1^, and the lowest quantification limit was limonene, which was 0.0029 μg·L^−1^.

##### Quantitative Results of Jasmine 

The volatile components of jasmine under different conditions were tested by quantitative analysis, and the results are shown in Supplementary Table S2. The kinds of volatile components remained the same during those treatments, but the amounts of each component were quite different. Those volatile components, including benzyl acetate, (E)-neryl alcohol, benzyl butyrate, α-farnesene, γ-yilanoleene, linalool, cis-3-hexenyl benzoate, n-hexyl benzoate, methyl 2-aminobenzoate, methyl benzoate, and trans-fophyl acetate, were all greater than 100 μg·L^−1^.

#### 2.3.2. Quantitative Analysis of the Volatile Components of Jasmine Tea

##### Establishment of Standard Curve of Jasmine Tea

During the scenting process, jasmine tea was collected every two hours during the whole experiment to serve as the QC samples. A quantity of 1000 g jasmine tea was weighed for the QC samples, in which the tea samples were powder samples. A blank matrix of jasmine tea from all samples was prepared to eliminate the volatile components. The standard curves of 28 volatile components were established under the same conditions (Supplementary Table S3). The R^2^ values were all between 0.9000 and 0.9999. Phenyl ethanol had a low response value; its linear range was the largest, ranging from 1.996 to 79,920.0000 μg·L^−1^, and its detection limit and quantification limit were also very high, 0.0674 μg·L^−1^ and 0.3923 μg·L^−1^, respectively. The linear range of limonene was the smallest, from 2.013 to 50.325 μg·L^−1^, respectively; the lowest detection limit was ethyl salicylate, 0.0006 μg·L^−1^, and the lowest quantification limit was limonene, 0.0030 μg·L^−1^.

##### Quantitative Results of Jasmine Tea

The quantitative detection results of the volatile components of jasmine tea were analyzed. The results are shown in Supplementary Table S4. The effective volatile components were the same during the different scenting processes, but there were some differences in each volatile component at different times. The volatile components, including phenethyl alcohol, benzyl benzoate, benzyl acetate, cis-3-hexenyl butyl, phytol acetate, methyl o-ethyl aminobenzoate, α-farnesene, cis-3-hexenoic acid cis-3-hexenyl ester, linalool, α-gingerene, and other compounds, were all greater than 100 μg·L^−1^.

### 2.4. Analysis of the Difference in Volatile Components of Jasmine and Jasmine Tea 

#### 2.4.1. Analysis of the Difference in Volatile Components of Jasmine 

##### PCA and HCA Analysis

**Figure 3:**
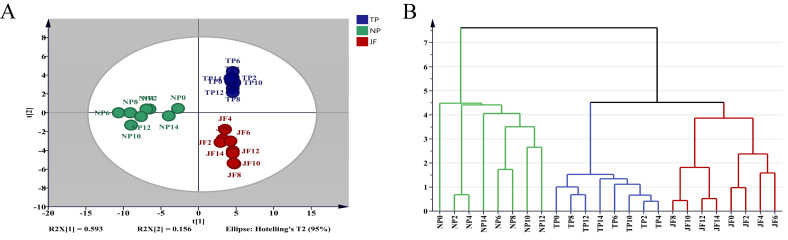
Comparison and analysis of volatile components in jasmine during the different scenting processes. (**A**) PCA score chart. (**B**) HCA chart.

Seven effective principal components were extracted by PCA (principal component analysis), which reached 97.5883% of the effective information on volatile components. In the fitting curve, the fit parameters R^2^ and Q^2^ were 0.956 and 0.887, respectively, which indicated that the model fitted well. The PCA score chart is shown in Figure 3A. The model could well distinguish jasmine under different treatments, and the model classification was completely consistent with the experimental classification. In the HCA (hierarchical cluster analysis) chart (Figure 3B), the samples could be divided into three categories which were completely consistent with the PCA chart and experimental classification. 

For potential differential volatile components, the following indicators were used to screen the differential volatile components at different stages of the scenting process: ① variable importance in projection (VIP) value was greater than 1; ② for each comparison, statistical significance was characterized by a fold change of 1.5 (ratio > 1.5 or < 0.67) and *p* < 0.05; in addition, the statistical S-plots of the OPLS-DA(discriminant analysis of orthogonal partial least squares) model and the corresponding jack-knifed confidence interval loading chart were used as an auxiliary tool for the identification of differential volatile components. Analysis of the Volatile Components between TP and NP

**Figure 4:**
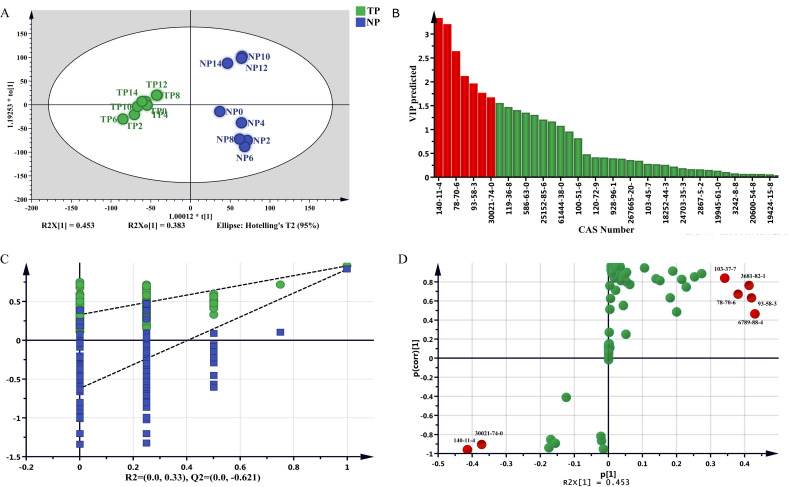
Comparative analysis of volatile components of TP and NP. (**A**) OPLS-DA score plot. (**B**) VIP plot. (**C**) permutation tests plot. (**D**) S-plot. The color in B and D means that differential volatile component is marker in green color; those significant differential volatile components with a VIP > 1 and FC > 1.5 or <0.67 (*p* < 0.05) marker in red color.

To explore the different volatile components of jasmine during different scenting processes, all jasmine samples under TP and NP were analyzed by OPLS-DA. As shown in Figure 4A, the statistical parameters were R^2^X(cum) = 0.983, R^2^Y(cum) = 0.945, and Q^2^(cum) = 0.908 in OPLS-DA analysis of NP and TP. In OPLS-DA analysis, the closer each value was to 1, the higher the predictability and applicability of the model. Figure 4C shows that both Hotelling’s T^2^ and D Mod X had no outliers within the 95% confidence range, and in 200 permutation tests, the intercept value of the model was significantly lower than that of the original model distance (R^2^ = 0.271, Q^2^ = −0.699). The results showed that the model was not overfitting. According to the VIP prediction (Figure 4B,D), there were 7 kinds of differential volatile component with a VIP > 1.0 and FC > 1.5 or < 0.67 (*p* < 0.05), including benzyl acetate, benzyl butyrate, linalool, α-farnesene, n-hexyl benzoate, (E)-3-hexen-1-acetate, and methyl benzoate. 

##### Analysis of the Volatile Components between JF and TP

**Figure 5:**
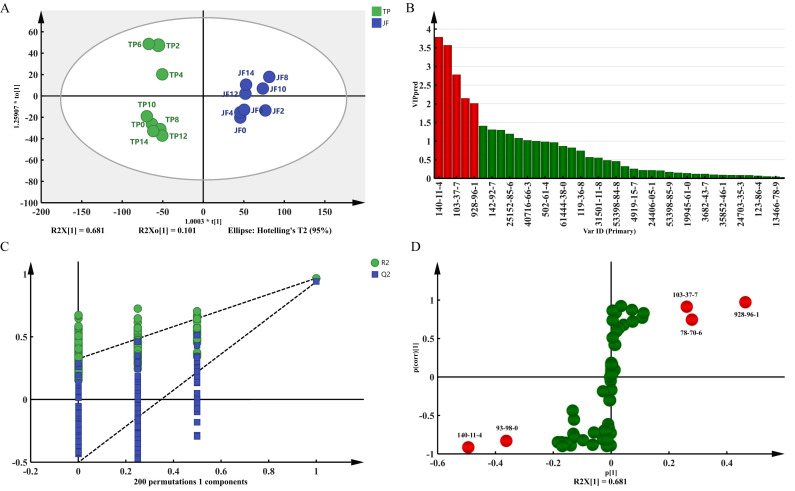
Comparative analysis of volatile components of JF and TP. (**A**) OPLS-DA score plot. (**B**) VIP plot. (**C**) permutation tests plot. (**D**) S-plot. The color in (**B**) and (**D**) means that differential volatile component is marker in green color; those significant differential volatile components with a VIP > 1 and FC > 1.5 or <0.67 (*p* < 0.05) marker in red color. Analysis of the Volatile Components between JF and NP.

The statistical parameters of the OPLS-DA model of JF and TP are shown in Figure 5A: R^2^X(cum) = 0.891, R^2^Y(cum) = 0.949, and Q^2^(cum) = 0.927. Figure 5C of the permutation test shows that Hotelling’s T^2^ and D Mod X had no outliers within the 95% confidence range. In 200 permutation tests, the intercept value of the model was significantly lower than that of the original model (R^2^ = 0.225, Q^2^ = −0.528). This result showed that this model did not appear to be overfitting. According to the VIP prediction (Figure 5B) and S-plot (Figure 5D), there were 5 kinds of differential volatile component, including ethyl benzoate, cis-3-hexanol, linalool, benzyl acetate, and benzyl butyrate.

**Figure 6:**
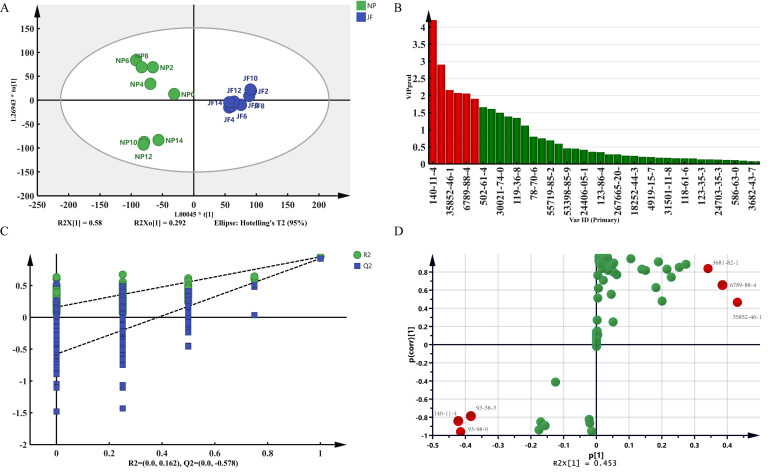
Comparative analysis of volatile components of JF and NP. (**A**) OPLS-DA score plot. (**B**) VIP plot. (**C**) permutation tests plot. (**D**) S-plot. The color in B and D means that differential volatile component is marker in green color; those significant differential volatile components with a VIP > 1 and FC > 1.5 or <0.67 (*p* < 0.05) marker in red color.

The statistical parameters of the OPLS-DA model of JF and NP are shown in Figure 6A: R^2^X(cum) = 0.972, R^2^Y(cum) = 0.950, and Q^2^(cum) = 0.936. There were no outliers in Hotelling’s T^2^ and D Mod X within the 95% confidence range (Figure 6C). In 200 permutation tests, the intercept value of the model was significantly lower than that of the original model (R^2^ = 0.106, Q^2^ = −0.484). According to the VIP prediction in Figure 6B and S-plot in Figure 6D, there were 6 kinds of differential volatile component, including benzyl acetate, methyl benzoate, valeric acid-(Z)-3-hexenyl ester, α-farnesene, 2-aminobenzoate, ethyl benzoate, and n-hexyl benzoate.

#### 2.4.2. Analysis of the Difference in Volatile Components of Jasmine Tea

**Figure 7:**
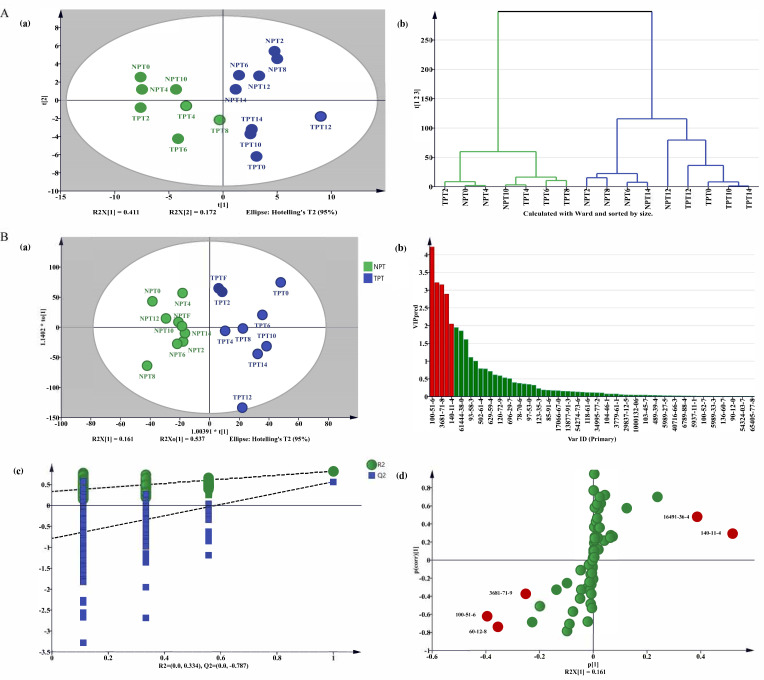
Comparative analysis of different volatile components of jasmine tea. (**A**) Comparison and analysis of volatile components in jasmine tea during the different scenting processes: (**a**) PCA score chart; (**b**) HCA chart. (**B**) Comparative analysis of volatile components of TPT and NPT: (**a**) OPLS-DA score plot; (**b**) VIP plot. (**c**) permutation tests plot. (**d**) S-plot. The color in (**B**)/(**b**) and (**d**) means that differential volatile component is marker in green color; those significant differential volatile components with a VIP > 1 and FC > 1.5 or <0.67 (*p* < 0.05) marker in red color.

In this study, the PCA model extracted 7 principal components among which the model fitting parameters R^2^ and Q^2^ were 0.969 and 0.949, respectively. From the PCA score chart (Figure 7A(a)), it could be concluded that the model could distinguish jasmine tea during different experimental treatments. The HCA chart (Figure 7A(b)) was completely consistent with the PCA chart and experimental classification.

Figure 7B(a) shows that the statistical parameters were as follows: R^2^X(cum) = 0.969, R^2^Y(cum) = 0.887, and Q^2^(cum) = 0.986. It could be seen from Figure 7B(c) showed that both Hotelling’s T^2^ and D Mod X had no outliers within the 95% confidence range. According to the VIP prediction in Figure 7B(b) and S-plot in Figure 7B(d), there were 5 kinds of differential volatile component during the different scenting processes, including benzyl alcohol, phytol acetate, phenethyl alcohol, cis-3-hexenyl butyl ester, and benzyl acetate.

### 2.5. Analysis of the Main Differential Volatile Components

To examine the effects of the different scenting processes on the aroma quality of jasmine and jasmine tea, changes in aroma volatiles during the scenting process were determined systematically. From Table 1, it could be seen that the volatile compounds of jasmine all showed a trend of rising first and then falling, except for benzyl butyrate esters and benzyl alcohol, which showed a downward trend. The main volatile components of jasmine tea rose first and then remained stable or slightly decreased during the scenting process. 

### 2.6. Changes in the Main Aroma Components of Jasmine and Jasmine Tea

The volatile components in jasmine and jasmine tea according to GC-MS analysis were mainly divided into terpenes, esters, alcohols, nitrogen-containing compounds, oxygen-containing compounds, and hydrocarbons in this study.

#### 2.6.1. Changes in Main Aroma Substances of Jasmine

**Figure 8:**
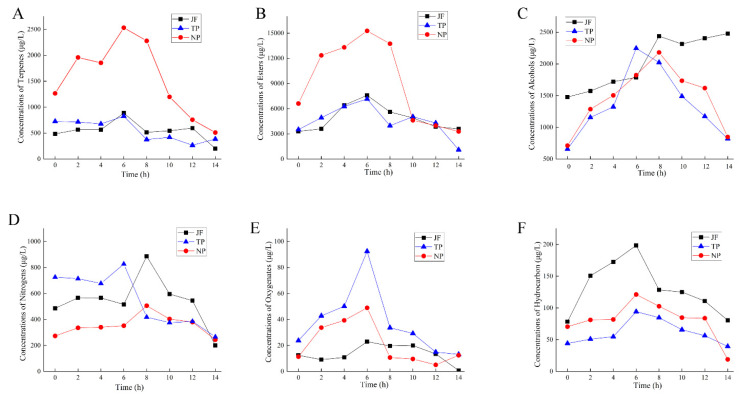
Changes in main aromatic substances of jasmine during the scenting process. (**A**) terpenes. (**B**) esters. (**C**) alcohols. (**D**) nitrogens. (**E**) oxygenates. (**F**) hydrocarbons.

As shown in Figure 8A, the amounts of terpenoids in NP reached a maximum of 2531.10 μg·L^−1^ at 6 h, while the JF and TP reached peaks at 6 h of 886.34 μg·L^−1^ and 826.07 μg·L^−1^, respectively. The changes in esters showed a trend of increasing and then decreasing (Figure 8B). The JF reached the maximum value of 7590.07 μg·L^−1^ at 6 h and the lowest value of 3852.80 μg·L^−1^ at 14 h. The jasmine of NP and TP reached the maximum value at 6 h, 15269.82 μg·L^−1^ and 7149.34 μg·L^−1^, respectively; the lowest values, at 14 h, were 3286.96μg^·L−1^ and 1102.07 μg·L^−1^, respectively. From Figure 8C, we could see that JF had the highest amounts of alcohol among those treatments. The TP reached the maximum value at 6 h, 2246.47 μg·L^−1^. The NP reached the maximum value at 8 h, 2180.61 μg·L^−1^. The changes in nitrogen-containing compounds showed a trend of increasing and decreasing during the scenting process (Figure 8D). The trends of nitrogen-containing compounds in JF and NP were similar; the highest values, at 8 h, were 886.84 μg·L^−1^ and 826.20 μg·L^−1^, respectively, and the lowest values, at 14 h, were 200.87 μg·L^−1^ and 244.54 μg·L^−1^. The nitrogen-containing compounds of TP reached a maximum value of 506.61 μg·L^−1^ at 6 h and a minimum value of 265.87 μg·L^−1^ at 14 h. As shown in Figure 8E,F, the amounts of oxygen compounds and hydrocarbons rose first and then fell during the scenting process. The maximum amount of oxygen compounds in TP was 92.46 μg·L^−1^ at 6 h. The maximum amount of hydrocarbons in JF was 198.51 μg·L^−1^, and the lowest amount in TP was 94.19 μg·L^−1^. 

#### 2.6.2. Changes in Main Aroma Substances of Jasmine Tea

**Figure 9:**
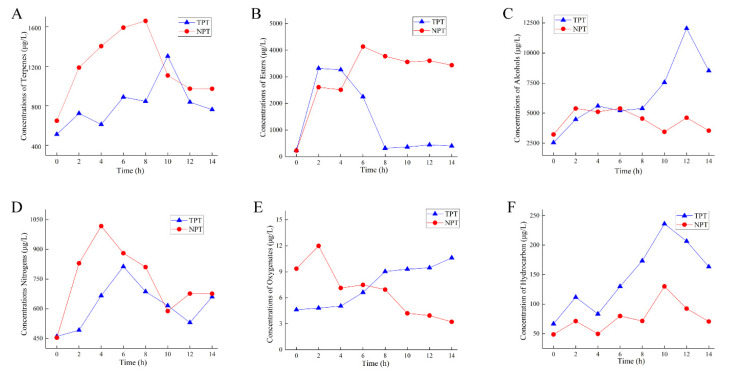
Changes in the main aromatic substances of jasmine tea during scenting. (**A**) terpenes. (**B**) esters. (**C**) alcohols. (**D**) nitrogens. (**E**) oxygenates. (**F**) hydrocarbons.

The changes in terpenoids in jasmine tea during the scenting process are shown in Figure 9A. The amounts of terpenoids in NPT increased to 1659.08 μg·L^−1^ at 8 h and then decreased at 12 h to 973.23 μg·L^−1^. The changes in esters showed a trend of increasing and then decreasing (Figure 9B). The amount of esters in NPT increased to 4137.96 μg·L^−1^ at 6 h and decreased to 3437.85 μg·L^−1^ at 14 h. The maximum value of TPT was 3319.03 μg·L^−1^ at 2 h, dropped to 318.34 μg·L^−1^ during 4–8 h, then remained balanced. The changes in alcohols are shown in Figure 9C. The amounts of alcohols in NPT increased to 5387.12 μg·L^−1^ at 2 h and decreased to 3446.69 μg·L^−1^ at 10 h. The amounts of TPT increased to 5559.35 μg·L^−1^ at 4 h, continued to rise to 12041.01 μg·L^−1^ at 12 h, and finally decreased to 8521.66 μg·L^−1^ at 14 h. The changes in nitrogen-containing compound amounts are shown in Figure 9D. The NPT increased to 1017.23 μg·L^−1^ at 4 h, decreased to 588.04 μg·L^−1^ at 10 h, rose to 676.42 μg·L^−1^ at 12 h, then remained stable. After 6 h of TPT, the tea rose to the maximum value of 812.79 μg·L^−1^ then dropped to 530.13 μg·L^−1^ within 12 h and increased to 659.66 μg·L^−1^ later. The changes in oxygenated compounds during the scenting process are shown in Figure 9E. The amounts of oxygenated compounds in tea in the new scenting process first increased and then decreased, while it showed a continuous upward trend during the traditional process. The tea under NPT increased to a maximum of 11.99 μg·L^−1^ after 2 h of scenting and then dropped to 3.22 μg·L^−1^ at 14 h. The TPT showed a rising trend, with a minimum value of 4.61 μg·L^−1^ and maximum value of 10.60 μg·L^−1^. From Figure 9F, the maximum value of hydrocarbons in the NPT was 129.96 μg·L^−1^, and the minimum value was 70.567 μg·L^−1^; the maximum value of TPT was 236.11 μg·L^−1^, and the minimum value was 66.63 μg·L^−1^.

## 3. Discussion

The aromas of jasmine and jasmine tea of different scenting processes during different times would be different. In this experiment, we analyzed the volatile compounds of those samples to explore the changes between jasmine and jasmine tea during different scenting processes.

The whole scenting process, including the aroma released in jasmine and the absorbability of tea dhool through both physical adsorption and chemical absorption, would be affected by the scenting process and environmental conditions, which could cause changes in compounds and volatiles [20,21]. The pile height was relatively high in the traditional scenting process, resulting in high temperature, which had a bad influence on jasmine. After the pile temperature reached a certain level in the traditional scenting process, the pile needed to be turned over in order to lose parts of the heat and moisture. Otherwise, the jasmine would lose water too fast and cause an unpleasant aroma. However, during the new scenting process, the pile temperature increased slowly and stayed at a relatively lower level, due to the thin pile height. In this study, the pile temperature of the new scenting process was closer to room temperature (25–28 °C), which could reduce the process of turning the pile, simplify the scenting process, and save labor. The humidity of JF was higher than that of scented jasmine and the environment. The humidity had a closer relationship with the water content. The water content of jasmine showed a trend of increasing and then decreasing. The water content of jasmine from buds to tiger paws increased with the strengthening of respiration. Then, the respiration of jasmine decreased as the degree of opening increased, and the water content decreased. Therefore, in the later opening period, the water content of jasmine was significantly lower than that in the early opening period. The tea dhool absorbed the aroma and water from jasmine during the scenting process, which caused the water content to decrease in jasmine and increase in tea dhool.

The water content is thought to play an important role during the scenting process [5]. The water content of the tea was related to the water produced by the respiration of jasmine. When the tea dhool contained a certain amount of water, it could better absorb aromatic substances and improve the quality of scented tea [22]. The adsorption capacity increases with the increase in the water content of the tea dhool within a limited range [23]. In addition to maintaining the vitality of jasmine, a higher water content is beneficial to the oxidation–reduction reaction between the aroma substances and the polyphenolic compounds in tea, thereby improving the adsorption effect [24]. Compared with the traditional scenting process, the new scenting process could better maintain the freshness of jasmine, extend the jasmine fragrance release time, and reduce the process of turning the temperature pile to the lower level, which could better reduce labor intensity and costs.

Aroma is an important factor in evaluating the quality of jasmine tea. By analyzing the changes in volatile substances in the scenting process, the technological parameters of jasmine tea scenting can be obtained.

The JTF index value will increase with the quality of jasmine and jasmine tea [7,25]. Therefore, the JTF index was chosen to describe the correlation between the volatile components and the quality of jasmine and jasmine tea. The rate of increase in the new scenting process was lower than that of the traditional scenting process. The pile height of the traditional process conditions was higher, the jasmine released the fragrance faster, the JTF index increased rapidly, and jasmine wilted faster. The JTF of jasmine and jasmine tea showed a trend of increase and decrease. JTF indices in NP and NPT were lower than TP and TPT before reaching the highest point, then showed an opposite change.

The volatiles of tea are present in relatively lower quantities (approximately 0.01% of the total dry weight) and play an important role in flavor because of the lower odor detection, especially in jasmine tea [26]. Most of the volatile components had a special fragrance type, which could give jasmine a special flavor. The changes in these substances were the main factors that could affect the fragrance type and concentration of jasmine. Volatile compounds of tea are classified into nonterpenoids (products of lipid oxidation), which impart undesirable grassy odors, and terpenoids (linalool and geraniol), which are mainly responsible for the sweet and flowery aroma of tea [26]. The major compounds of jasmine tea were linalool, benzyl acetate, benzyl alcohol, methyl anthranilate, indole, etc. [27]. Researchers thought that nerolidol and methyl benzoate were related to “overall”, linalool and methyl anthranilate to “jasmine”, and decanal and benzene acetaldehyde to “sweet floral”,“roasted”, and “fermented” [9]. Benzyl acetate, (Z)-3-hexenyl benzoate, linalool, benzyl alcohol, α-farnesene, methyl anthranilate, and indole contribute to favorable flowery and fruity aromas; nerolidol is divided into fruity flowers, and germacrene D has a woody smell [27,28]. There were: 7 kinds of differential volatile component in TP and NP, including benzyl acetate, benzyl butyrate, linalool, α-farnesene, n-hexyl benzoate, (E)-3-hexen-1-acetate, and methyl benzoate; 5 kinds of differential volatile component in JF and TP, including ethyl benzoate, cis-3-hexanol, linalool, benzyl acetate, and benzyl butyrate; and 6 kinds of differential volatile component in JF and NP, including benzyl acetate, methyl benzoate, valeric acid-(Z)-3-hexenyl ester, α-farnesene, 2-aminobenzoate, ethyl benzoate, and n-hexyl benzoate. There were 5 kinds of differential volatile component in jasmine tea during the different scenting processes, including benzyl alcohol, phytol acetate, phenethyl alcohol, cis-3-hexenyl butyl ester, and benzyl acetate.

Lu J [29,30] used HS-SPME combined with GC-MS to determine the content of volatile substances in jasmine tea during traditional scenting and isolation scenting and found that the main volatile substances in the scenting process were olefins, esters, alcohols, nitrogen compounds, and ketones. In the traditional scenting process, tea dhool should be baked before being next scented to ensure the water content is at a lower level [9]. One researcher suggested that a high level of water content of tea dhool could preserve the ecological conditions of jasmine and had a good effect on the aroma concentration and freshness of jasmine tea; this is called continuous wet scenting technology [31,32]. However, our results were different from them. In this study, we found that the jasmine tea produced by the traditional scenting process had a relatively lower quality than that produced by the new scenting process because of the higher level of water content. Removing the calyx and peduncle will promote the innovative use of jasmine flowers to maximize the release of aroma [33]. Another study showed that the process of jasmine fragrance release was the physiological movement of flower petal cells [34,35]. Ye NX et al. [23] found that cryopreservation could delay the rate of opening. The buds of jasmine were difficult to open in vitro when the ambient temperature was lower than 20 °C. However, when the room temperature is higher than 36 °C, the opening time of the flowers is advanced. Zhang LX et al. [36] analyzed the fragrance of jasmine on sunny and rainy days and found that the climate has a great influence on the composition of jasmine fragrance. Under traditional process conditions, the kinds of volatile components increased, among which the increase in ester compounds was the most significant. The change in volatile components in the new scenting process was relatively stable compared to that in the traditional scenting process, because the pile temperature, enzyme activity, and respiration rate in the new scenting process were lower than those in the traditional process. The amounts of volatile components in the new process (tea and jasmine) were higher than those in the traditional process. Compared with the traditional scenting process, the new process can obtain better quality without repeated scenting and drying. Therefore, the new process could better reduce the loss of aroma in the tea body. These results indicated that jasmine tea produced by the new scenting process had better volatile quality, which can provide proof for the new scenting process.

## 4. Materials and Methods

### 4.1. Materials

#### 4.1.1. Plant Material

*J. sambac* (L.) jasmine in this experiment was provided by Yunbiao Town, Hengxian, Guangxi, China. The green tea dhool used for jasmine tea was Shimen Yinfeng produced by one bud and one leaf in spring.

#### 4.1.2. Reagents 

Sodium chloride (analytical purity, Sinopharm Chemical Company, China); cis-3-hexenol (≥98%), linalool (≥99%), phenethyl alcohol (≥98.5%), geraniol (≥99%), (E)-nerolidol (≥99%), β-cyclic citral (≥98%), myrcene (≥98.5%), phytol acetate (≥97%), methyl benzoate (≥98%), benzyl acetate (≥97%), ethyl benzoate (≥97.5%), cis-3-hexenyl butyl ester (≥99%), 2-methylbutyrate folate (≥99%), juniperene (≥99%), phenethyl acetate (≥99%), ethyl salicylate (≥98%), methyl 2-aminobenzoate (≥98%), butyl benzyl acetate (≥97%), benzyl acetate (≥80%), cis-3-hexenoic acid cis-3-hexenate (≥96%), methyl o-methyl aminobenzoate (≥97%), cis-3-hexenyl butyl ester (≥99%), n-hexyl benzoate (≥97.5%), indole (≥98%), eugenol (≥96%), myrcene (≥79%), limonene (≥98%), 2-methylnaphthalene (≥96%), 1-methylnaphthalene (≥95%), β-caryophyllene (≥94%) (Beijing Bailingwei Technology Co., Ltd., China); benzene methanol (≥98.5%), benzaldehyde (≥99.5%), methyl salicylate (≥97.5%), ethyl decanoate (Sigma-Aldrich Reagent Company, Burlington, MA, USA); C8–C40 even-numbered n-alkane standard (o2si company, Charleston, SC, USA).

#### 4.1.3. Instrumentation

High-speed multifunctional pulverizer (LDP-350, Red Sun, Yongkang, Zhejiang, China); manual sampling handle for solid phase microextraction (SPME, Supelco, Bellefonte, PA, USA); digital display magnetic heating stirrer (EPFO-984TA7CHSEUA, Troemner(Talboys), Philadelphia, PA, USA); vacuum freeze dryer (Alpha 1-4/LSC Plus, Christ, Osterode, Germany); GC-MS (7890B-7000C, Agilent, Palo Alto, Santa Clara, CA, USA); vertical ultra-low temperature refrigerator (U410-86, Eppendorf, Hamburger, Germany); PDMS/DVB solid phase microextraction head (65 μm, Supelco, Bellefonte, PA, USA); electronic balance (AE240, Mettler Toledo, Zurich, Switzerland); glass test tube (50 mL, Shu Niu Company, Sichuan, China); headspace bottles (50 mL and 15 mL, Ampu Scientific Instruments, Shanghai, China); ultrapure water system (Z00QSV001, Millipore, Burlington, MA, USA).

### 4.2. Process Parameters of Different Scenting Processes of Jasmine Tea

After the jasmine teas were delivered to the laboratory, the scenting process was initiated when 70–80% of the jasmine was opened as tiger claw. The traditional process (TP) parameters referred to Chen MC et al. with one scenting [5]; the new process (NP) parameters referred to An HM et al. [18,19].

### 4.3. Optimization of Aroma Extraction Conditions

The conditions to extract the volatile components of jasmine and jasmine tea referred to the methods of Lin et al. [37].

#### 4.3.1. HS-HPME Extraction Process of Jasmine

Improvement of the extraction device: the extraction device was redesigned and manufactured to retain the jasmine aroma, which can ensure that the aroma is fully volatilized and that ethyl decanoate could be released in the same environment at a better and uniform speed.

**Figure 10:**
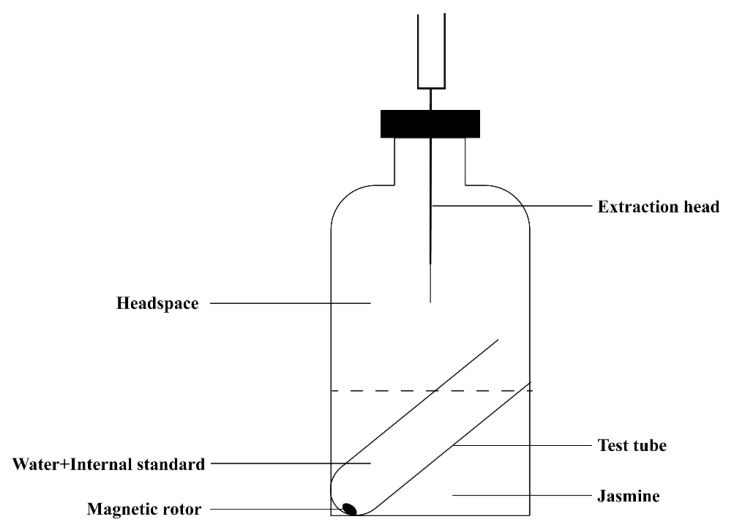
Diagram of aroma extraction device.

Jasmine extraction: 5 g of jasmine was placed in a 250 mL headspace bottle; 5 mL of deionized water, 20 μL of ethyl decanoate (8.64 mg·L^−1^), and a magnetic rotor (1 × 0.5 × 0.5 mm) were added to the test tube. The test tube was placed in the headspace bottle, and the headspace bottle cap was closed tightly (Figure 10). This was placed on a magnetic heating stirrer with the parameters set to 800 rpm·min^−1^ and equilibrated at 40 °C for 5 min, then extracted for 30 min. The solid-phase microextraction head was inserted into the headspace part of the headspace bottle and equilibrated for 5 min and extracted for 40 min. The fiber extraction head was retracted after the extraction was completed and then inserted into the GC, fully eluted at 230 °C for 5 min, and subjected to GC-MS detection.

#### 4.3.2. HS-HPME Extraction Process of Jasmine Tea

Extraction: 1 g of jasmine tea powder was placed in a 15 mL headspace bottle; a magnetic rotor, 5 mL boiled deionized water, and 0.5 g sodium chloride were added to fully dissolve the sample, and then 10 μL ethyl capric acid ester (8.64 μg·L^−1^) was added to the headspace bottle. This was placed on a magnetic heating stirrer, with the parameters set to 800 rpm·min^−1^ and 40 °C. The solid-phase microextraction head was inserted into the headspace part of the headspace bottle, equilibrated for 10 min, and extracted for 40 min. The fiber extraction head was retracted after the extraction was completed, inserted into the GC, fully eluted at 230 °C for 5 min, and then subjected to GC-MS detection.

#### 4.3.3. Analysis of GC-MS Condition

GC: chromatographic column HP-5MS (30 m × 0.25 mm × 0.25 μm); carrier gas was helium (purity > 99.999%); inlet temperature was 250 °C; the column flow rate was 1 mL·min^−1^; split ratio: 5:1; initial temperature: 50 °C kept for 3 min, increased at 4 °C·min^−1^ to 100 °C, kept for 1 min; increased at 6 °C·min^−1^ to 180 °C; increased at 16 °C·min^−1^ to 230 °C; solvent delayed 3 min.

MS: ion source temperature: 230 °C; ion source: EI; EI source energy: 70 eV; interface temperature: 250 °C; electron multiplier voltage: 1104 V; using full scan mode (scan) scanning range: 35~400 amu.

### 4.4. Qualitative Analysis Data Processing Methods

Qualitative method: ① We retrieved and calibrated the gas chromatographic information of each gas chromatographic peak obtained from the detection results of GC-MS, using the NIST 17.0 standard spectrum database, and selected the result with a matching degree greater than 80% as the identification standard for the substance. We measured the retention time of the substance, obtained the retention index (RI) of the substance through the retention index calculation formula, and proofread and appraised it by combining the retention index in the literature and the database.

(2) $$\mathrm{RI}=100x+100\times \left[\frac{\mathrm{RTm}-\mathrm{RTx}}{\mathrm{RT}\left(x+1\right)-\mathrm{RTx}}\right]$$

In the formula: RI, retention index of the substance to be tested; RT, retention time of the substance to be tested; RTx, retention time of normal alkanes with a number of carbon atoms; RT(x + 1) − RTx, the retention time of n-alkanes with the number of carbon atoms.

### 4.5. Establishment of Quantitative Method

In this experiment, the opening degree and aroma of the jasmine flowers of jasmine and green tea dhools at different times during the scenting process were different. To improve the scientific accuracy, the results were compared with the aroma substance library for calibration and verification after GC-MS analysis to determine the composition and content of several ingredients of jasmine and jasmine tea.

#### 4.5.1. Preparation of Blank Matrix

First, 300 g of QC sample was weighed and transferred to a 1 L round bottom flask, then 300 mL of ultrapure water was added. The rotary evaporator was turned on, andwater was continually replenished at low temperature (40–55 °C). Rotation and evaporation were performed regularly, and samples were tested regularly until the signal-to-noise ratio < 3, then freeze-dried as the blank matrix of the samples [15].

#### 4.5.2. Establishment of a Standard Curve

Extraction conditions of jasmine: we drew a certain amount of mixed standard, added an appropriate amount of chromatographic pure absolute ethanol, configured it into mother liquor, and diluted the mother liquor to 20,000, 10,000, 5000, 2500, 1250, 625, 500, 250, 100, 50, 25, 5, and 1—thirteen different concentrations of mixed standards. 

Extraction conditions of tea: we drew a certain amount of mixed standard, added an appropriate amount of chromatographic pure absolute ethanol, configured it into mother liquor, and diluted the mother liquor to 20,000, 10,000, 5000, 2500, 1250, 625, 500, 250, 100, 50, 25, 5, and 1—thirteen different concentrations of mixed standards. Then, 10 μL of the mixed standard was added to a headspace bottle containing 1 g blank matrix and 0.5 g sodium chloride. A magnetic rotor was added; 5 mL of boiling ultrapure water was added; and, finally, 20 μL ethyl caprate (8.64 mg·L^−1^) was added.

Standard curve drawing: HS-SPME extraction and GC-MS analysis were performed when the parameters were consistent with the sample detection parameters. The concentration of each standard was taken as the abscissa, and the ratio of the peak area of the standard to the internal standard was taken as the ordinate. Each concentration was repeated 3 times to establish a standard curve. At the same time, the quantification limit LOQ (limit of quantitation) and the detection limit LOD (limit of detection) of each substance were detected under this method. According to the standard curve, the corresponding volatile components were quantitatively analyzed. For compounds without standard products, standard products with the same functional group and similar carbon number were used for estimation.

### 4.6. Calculation of Aroma Activity Threshold (OAV)

OAV was the ratio of the concentration of a certain aroma component to its olfactory threshold. We found the relevant literature [38,39] to obtain the threshold and then calculated the OAV value based on the quantitative results. The calculation formula was as follows:

(3) $$\mathrm{OAV}=\frac{\mathrm{Ci}}{\mathrm{OTi}}$$

In the formula, Ci is the concentration of the aroma component (μg·L^−1^), and OTi is the threshold value of the aroma component (μg·L^−1^) [40]. 

### 4.7. Data Processing

The Agilent Mass Hunter 7.0 workstation was used for qualitative and quantitative analysis of aroma components; SPSS 23.0 was used for variance analysis and multiple regression analysis,;and SIMCA-P 14.1 was used for multiple statistical analysis.

## 5. Conclusions

There were 71 kinds of effective volatile component in jasmine, including 9 alcohols, 24 esters, 24 terpenes, 6 hydrocarbons, 1 ketone, 3 aldehydes, 2 nitrogen-containing compounds, and 2 oxygen-containing compounds. A total of 78 effective volatile components were detected in jasmine tea, including 6 alcohols, 28 esters, 29 terpenes, 8 nitrogen-containing compounds, 1 aldehyde, and 6 others compounds.

The JTF of jasmine and jasmine tea showed a trend of first increasing and then decreasing during the scenting process. Compared with the traditional scenting process, the change in the JTF index of jasmine during the new scenting process was relatively small, and the overall maintenance was at a high level, which was closer to the changing trend of the JTF index of isolated jasmine. 

The quantitative results showed that the kinds of volatile components of jasmine did not change significantly during different periods, but the amounts of each volatile component were quite different. The amounts of volatile components of jasmine in terpenes, oxygenates, esters, alcohols, nitrogen compounds, and hydrocarbons all showed a trend of increasing first and then decreasing. The alcohols and hydrocarbons of TP were higher than those of NP. The volatile components of jasmine tea generally increased first and then decreased. The amounts of volatile components of jasmine tea in terpenes, esters, alcohols, nitrogen-containing compounds, and hydrocarbons all increased first and then decreased. The oxygenated compounds in the NPT first rose and then fell, while the TPT showed an upward trend. Except for the amounts of alcohols and hydrocarbons in the TPT, other volatile components were lower than those in the NPT.

The quantitative results obtained were analyzed by PCA, HCA, and OPLS-DA, and substances with a VIP value greater than 1 were selected and summarized as differential volatile components, namely, benzyl butyrate, benzyl acetate, n-hexyl benzoate esters, methyl benzoate, ethyl benzoate, 3-hexen-1-ol, phyllo acetate, α-farnesene, linalool, cis-3-hexanol, and benzyl alcohol. During the scenting process, the main volatile components of jasmine tea first increased and then remained stable or slightly decreased. The benzyl butyrate and benzyl alcohol of scented flowers and isolated fresh flowers showed a downward trend. Other volatile components all showed a trend of rising first and then falling. The amounts of volatiles in jasmine and tea produced by the new scenting process were higher than that of the traditional scenting process at the same time. These results indicated that jasmine tea produced by the new scenting process had better volatile quality, which can provide proof for the new scenting process.

## Figures and Tables

**Table 1:** Changes in differential volatile components.

No.	Time	Benzyl Butyrate	Benzyl Acetate	N-Hexyl benzoate	Methyl Benzoate	Ethyl Benzoate	Methyl 2-Aminobenzoate	Phytol Acetate	α- Farnesene	Linalool	Cis-3-hexanol	Benzyl Alcohol
JF	0	—	↑	↑	↑	—	↑	↑	—	—	↑	↑
	2	↓	↑	↑	↓	↑	↑	↑	↑	↑	↑	↓
	4	↓	↓	—	↓	↑	↓	↓	↑	↓	↑	↓
	6	↓	↓	↑	↓	—	↓	↓	↑	↓	↑	↓
	8	↓	↓	↑	↓	↑	↓	↓	↑	↓	↑	—
	10	—	↓	↑	—	↓	—	↓	↓	↓	↓	—
	12	—	↓	↑	↓	↓	↓	↓	↓	↓	↓	—
	14	—	↓	↑	↓	↓	↓	—	↓	↓	↓	—
NP	0	↑	↑	↑	↑	↑	↑	/	↑	↑	↑	↑
	2	↑	↑	↑	↑	↑	↑	/	↑	↑	↑	↑
	4	↑	↑	↑	↓	—	↑	/	↑	↑	↑	↓
	6	↓	↑	↓	—	↑	↑	/	↑	↑	↑	↓
	8	↓	↓	↓	↓	↓	↓	/	↓	↑	↓	—
	10	↓	↓	—	↓	↓	↑	/	↓	↑	↓	—
	12	↓	↓	—	↓	↓	↓	/	↓	↑	↓	—
	14	↓	↓	—	↓	↓	↓	/	↓	↑	↓	↓
TP	0	↑	↑	↑	↑	↑	↑	↑	↑	↓	↑	↓
	2	↑	↑	—	↑	↑	↑	↑	↑	↓	—	↓
	4	↑	—	↑	—	—	↑	↑	—	↓	↑	—
	6	↑	↑	↑	↑	↑	↑	↑	↑	—	↑	↓
	8	↓	↓	↓	—	—	↑	—	↓	↓	—	↓
	10	↓	↓	↓	—	↑	↑	↑	↓	—	↑	↓
	12	↓	—	↓	—	↓	—	↑	—	—	↑	↓
	14	—	—	↓	↓	↓	↓	↓	—	↓	↓	↓
NPT	0	—	—	—	—	—	—	—	—	—	/	—
	2	↑	↑	↑	↑	↑	↑	↑	↑	↑	/	↑
	4	↑	—	↑	↑	—	↑	↑	—	↑	/	—
	6	↑	↑	—	↑	↑	↑	↑	—	↑	/	↑
	10	—	↑	—	↑	—	↑	↑	—	—	/	—
	12	↑	↓	—	↑	—	↑	↑	↓	↓	/	—
	14	↓	—	—	↓	—	↑	↑	—	↓	/	↑
	F	↓	—	—	↓	↑	—	↓	↓	—	/	↓
TPT	0	—	—	—	↑	—	—	—	↑	↑	/	↑
	2	↑	↑	↑	—	↑	↑	—	↓	↓	/	↑
	4	↑	↑	↑	—	↑	↑	↑	↓	↓	/	↑
	6	↑	—	—	—	↑	↑	↑	↓	↓	/	↑
	8	↑	—	↑	—	↑	↑	↑	↓	↓	/	↓
	10	↓	—	↑	—	↑	↑	↑	↓	↓	/	↓
	12	↓	↓	↑	↓	↑	↑	↓	↓	↓	/	↓
	14	↓	↓	↓	↓	↑	↓	↓	↓	↓	/	↓

Note: —: the compound showed a steady trend during this period. /: the compound had no quantitative results during this period. ↑ or ↓: indicating that this compound showed an upward or downward trend during this period.

## Data Availability

Not applicable.

## Supplementary Materials

Table S1: The qualitative and quantitative analysis of volatile components of jasmine flower; Table S2: The quantitative results of volatile constituents of jasmine; Table S3: The qualitative and quantitative analysis of volatile components of jasmine tea; Table S4: The quantitative results of volatile constituents of jasmine tea; Table S5: The variations of OAV values of aroma components in jasmine flower and jasmine tea; Figure S1. The main identified compounds of jasmine in TIC; Figure S2. The main identified compounds of jasmine tea in TIC.

## Sample Availability

Samples are not available from authors.

## Abbreviations

JF	Jasmine flower
NP	Jasmine flower in new scenting process
TP	Jasmine flower in traditional scenting process
NPT	Jasmine tea in new scenting process
TPT	Jasmine tea in traditional scenting process
En	Environment
JTF	Jasmine tea flavor index
RI	Retention index
QC	Quality control
LOQ	Limit of quantitation
LOD	Limit of detection
PCA	Principal component analysis
HCA	Hierarchical cluster analysis
OPLS-DA	Discriminant analysis of orthogonal partial least squares
