# Supplementary material for: Analysis of Volatile Components of Jasmine and Jasmine Tea during Scenting Process
Source: Molecules. 2022 Jan 12;27(2):479. doi: 10.3390/molecules27020479 (PMC8779377; doi:10.3390/molecules27020479)
Supplement: Supplementary file 1 [file molecules-27-00479-s001.zip › molecules-1520825-supp.mat.pdf]

Supplementary Table

Table S1 The qualitative and quantitative analysis of volatile components of jasmine flower

No.	a. RI	Compound	b. Identification method	Linear equations	R	Linear range(μg/L)	LOD (μg/L)	LOQ (μg/L)
15	1396	(1S,4S,4aS)-1-Isopropyl-4,7-dimethyl-1,2,3,4,4a,5-hexahydronaphthalene	MS	y = 0.014x + 18.9259	0.994	2.033-20330.003	0.283	0.8556
15	1116	(E)-4,8-Dimethylnona-1,3,7-tRIene	MS	y = 0.014x + 18.9258	0.994	2.033-20330.002	0.283	0.8556
13	1352	alpha-Cubebene	MS RI	y = 13.409x - 0.2456	0.994	2.004-400.801	0.0029	0.0087
22	1518	alpha-Farnesene	MS RI	y = 0.3317x - 0.1144	0.994	22.014-2517.500	0.0356	0.0976
16	1492	alpha-Guaiene	MS	y = 2.5452x + 7.6260	0.995	2.001-20010.001	0.0298	0.1041
17	1482	gamma-Muurolene	MS RI	y = 1.1718x - 0.1008	0.991	2.003-1251.875	0.0019	0.0058
7	1646	tau-Cadinol	MS RI	y = 5.2054x - 3.3048	0.996	2.035-40700.001	0.3182	0.9242
9	1240	1H-Tetrazol-5-amine	MS	y = 13.709x - 18.391	0.991	1.996-79920.002	0.3842	1.1672
19	910	2-Methylbut-2-en-1-yl acetate	MS	y = 0.0686x + 78.4541	0.995	2.013-10065.000	0.0312	0.0144
9	1300	2-Phenylethyl tiglate	MS	y = 13.709x - 18.390	0.991	1.996-79920.001	0.3842	1.1672
19	1046	3-Carene	MS	y = 0.0686x + 78.4543	0.995	2.013-10065.002	0.0312	0.0144
8	926	3-Hexenoic acid, methyl ester	MS RI	y = 1.5029x -4.1255	0.997	2.011-5027.500	0.0124	0.0466
11	1499	3-Pyridinecarbonitrile, 1,4-dihydro-1-methyl-	MS	y = 8.2717x + 0.3220	0.997	2.001-20010.003	0.0355	0.1236
11	1267	5-Hexen-3-one	MS	y = 8.2717x + 0.3221	0.997	2.001-20010.004	0.0355	0.1236
18	< 800	Acetic acid, butyl ester	MS RI	y = 79.247x - 179.83	0.998	2.013-10065.000	0.0312	0.0941
18	1011	Acetic acid, hexyl ester	MS	y = 79.247x - 179.84	0.998	2.013-10065.001	0.0312	0.0941
15	1446	Aromandendrene	MS RI	y = 0.014x + 18.9256	0.994	2.033-20330.000	0.283	0.8556
9	1140	Benzene, 1-isocyano-3-methyl-	MS	y = 13.709x - 18.392	0.991	1.996-79920.003	0.3842	1.1672
17	1035	Bicyclo [3.1.0] hex-2-ene, 2-methyl-5-(1-methylethyl)-	MS	y = 1.1718x - 0.1009	0.991	2.003-1251.875	0.0019	0.0058
5	1539	Bisabolol	MS	y = 0.2311x - 0.5322	0.997	10.000-100098.001	0.0461	0.1392
8	1190	Butanoic acid, 3-hexenyl ester, (E)-	MS	y = 1.5029x -4.1257	0.997	2.011-5027.502	0.0124	0.0466
12	992	Butanoic acid, butyl ester	MS	y = 58.6021x+0.2183	0.995	1.986-198.602	0.001	0.0032
12	< 800	Butanoic acid, ethyl ester	MS RI	y = 58.6021x+0.2181	0.995	1.986-198.600	0.001	0.0032
12	1348	Butanoic acid, phenylmethyl ester	MS	y = 58.6021x+0.2182	0.995	1.986-198.601	0.001	0.0032
6	1374	Butyl benzoate	MS RI	y = 10.0408x + 36.2417	0.998	2.003-20030.000	0.1649	0.5121
8	1542	Cubenene	MS RI	y = 1.5029x -4.1256	0.997	2.011-5027.501	0.0124	0.0466
18	1339	Cyclohexane, 1-ethenyl-1-methyl-2-(1-methylethenyl)-4-(1-methylethylidene)-	MS	y = 79.247x - 179.86	0.998	2.013-10065.003	0.0312	0.0941
11	1393	Cyclohexane, 1-ethenyl-1-methyl-2,4-bis(1-methylethenyl)-, [1S-(1alpha, 2beta, 4beta)]-	MS	y = 8.2717x + 0.3218	0.997	2.001-20010.001	0.0355	0.1236
17	1086	Cyclohexene, 3-methyl-6-(1-methylethylidene)-	MS	y = 1.1718x - 0.1010	0.991	2.003-1251.875	0.0019	0.0058
21	1546	Naphthalene, 1,2,4a,5,6,8a-hexahydro-4,7-dimethyl-1-(1-methylethyl)-, [1S-(1alpha,4a beta,8a alpha)]-	MS RI	y = 4.5019x + 2.1466	0.998	10.000-100098.001	0.0461	0.1392
15	1432	(1R,2S,6S,7S,8S)-8-Isopropyl-1-methyl-3-methylenetricyclo [4.4.0.02,7] decane-rel-	MS	y = 0.014x + 18.9260	0.994	2.033-20330.004	0.283	0.8556
23	1502	(1S,2E,6E,10R)-3,7,11,11-Tetramethylbicyclo [8.1.0] undeca-2,6-diene	MS	y = 0.1845x + 0.1031	0.997	2.013-10065.000	0.0312	0.0921
23	1289	n-Valeric acid cis-3-hexenyl ester	MS	y = 0.1845x + 0.1031	0.997	2.013-10065.000	0.0312	0.0921
23	1019	Pentanoic acid, 4-methyl-2-oxo-, methyl ester	MS	y = 0.1845x + 0.1031	0.997	2.013-10065.000	0.0312	0.0921
11	1373	s-Triazolo[4,3-a] pyridine, 3,5,7-trimethyl-	MS	y = 8.2717x + 0.3219	0.997	2.001-20010.002	0.0355	0.1236
2	1504	α-Muurolene	MS RI	y = 0.081x +0.9257	0.998	2.023-101.151	0.0025	0.0072
5	1528	1,2,3,5,6,8a-hexahydro-4,7-dimethyl-1-(1-methylethyl)	MS std	y = 25.6214x +1.4573	0.994	2.013-50.326	0.001	0.0029

22	1558	(Z, E)-alpha-Farnesene	MS	RI	std	y = 0.3317x - 0.1144	0.994	22.014-2517.500	0.0356	0.0976
2	985	beta-Myrcene	MS	RI	std	y = 0.081x +0.9256	0.998	2.023-101.150	0.0025	0.0072
24	1568	1,6,10-Dodecatrien-3-ol, 3,7,11-trimethyl-, (E)-	MS	RI	std	y = 4.1012x + 0.4521	0.99	2.014-2517.500	0.0356	0.0976
20	1406	1H-Cycloprop[e]azulene, 1a,2,3,4,4a,5,6,7b-octahydro-1,1,4,7-tetramethyl-, [1aR-(1a alpha, 4 alpha,4a beta,7b alpha)]-	MS	RI	std	y = 1.7589x - 2.3431	0.996	2.024-202.400	0.0063	0.0182
1	806	3-Hexen-1-ol, (Z)-	MS		std	y = 0.5003x + 0.0624	0.99	2.039-1274.375	0.0016	0.0043
3	1005	3-Hexen-1-ol, acetate, (E)-	MS	RI	std	y = 42.428x - 70.243	0.99	10.020-5010.000	0.0024	0.0065
23	1566	3-Hexen-1-ol, benzoate, (Z)-	MS	RI	std	y = 0.1845x + 0.1031	0.997	2.013-10065.000	0.0312	0.0921
13	1259	Acetic acid, 2-phenylethyl ester	MS	RI	std	y = 13.409x - 0.2455	0.994	2.004-400.800	0.0049	0.0087
9	1172	Acetic acid, phenylmethyl ester	MS		std	y = 13.709x - 18.389	0.991	1.996-79920.000	0.3842	1.1672
14	1272	Benzoic acid, 2-hydroxy-, ethyl ester	MS	RI	std	y = 0.7502x + 2.1163	0.998	2.024-202.400	0.0063	0.0182
10	1174	Benzoic acid, ethyl ester	MS	RI	std	y = 1.3401x + 0.1546	0.991	2.042-25552.500	0.0049	0.0142
6	1094	Benzoic acid, methyl ester	MS	RI	std	y = 10.0408x + 36.2418	0.998	2.003-20030.001	0.1649	0.5121
5	1031	Benzyl alcohol	MS		std	y = 0.2311x - 0.5321	0.997	10.000-100098.	0.0461	0.1392
8	1143	Butanoic acid, 3-hexenyl ester, (Z)-	MS	RI	std	y = 1.5029x -4.1256	0.997	2.011-5027.501	0.0124	0.0466
21	1422	Caryophyllene	MS		std	y = 4.5019x + 2.1465	0.998	10.000-100098.	0.0461	0.1392
19	1390	cis-3-Hexenyl cis-3-hexenoate	MS		std	y = 0.0686x +78.4542	0.995	2.013-10065.001	0.0312	0.0144
12	1236	cis-3-Hexenyl-alpha-methylbutyrate	MS	RI	std	y = 58.6021x+0.2181	0.995	1.986-198.600	0.001	0.0032
17	1377		MS			y = 1.1718x - 0.1007	0.991	2.003-1251.875	0.0019	0.0058
4	1024	D-Limonene	MS	RI	std	y = 25.6214x +1.4572	0.994	2.013-50.325	0.001	0.0029
18	1383	Hexanoic acid, 3-hexenyl ester, (Z)-	MS	RI	std	y = 79.247x - 179.85	0.998	2.013-10065.002	0.0312	0.0941
7	1105	Linalool	MS	RI	std	y = 5.2054x - 3.3047	0.996	2.035-40700.000	0.3182	0.9242
16	1343	Methyl anthranilate	MS	RI	std	y = 2.5452x + 7.6259	0.995	2.001-20010.000	0.0298	0.1041
11	1195	Methyl salicylate	MS		std	y = 8.2717x + 0.3217	0.997	2.001-20010.000	0.0355	0.1236
15	1297	indole	MS	RI	std	y = 0.014x + 18.9257	0.994	2.033-20330.001	0.283	0.8556

Note: a. RI: Retentation index of compounds on HP-5MS. b. Method of identification: MS, Comparison of mass spectra with MS libraries and retention times. RI, retention index. Std, confirmed by authntic standards. LOD: Limit of detection. LOQ: Limit of quantitation.

Table S2 The quantitative results of volatile constituents of jasmine

Compounds	The quantitative of volatile components of the traditional scenting process jasmine								The quantitative of volatile components of the new scenting process jasmine								The quantitative of volatile components of the jasmine							
	0h	2h	4h	6h	8h	10h	12h	14h	0h	2h	4h	6h	8h	10h	12h	14h	0h	2h	4h	6h	8h	10h	12h	14h
3-Hexen-1-ol, acetate, (E)-	104.63	174.65	226.81	402.89	305.99	491.22	226.10	504.48	348.77	9.82	9.60	10.56	9.50	10.99	10.75	7.53	5.43	5.42	2.64	3.79	4.04	3.73	2.49	2.64
Benzoic acid, methyl ester	140.83	338.44	321.21	240.81	92.29	128.76	58.68	65.68	469.42	10.01	9.29	15.22	13.70	15.17	15.05	10.54	1.06	1.17	0.51	0.72	LOD	LOD	LOD	LOD
Acetic acid, phenylmethyl ester	######	######	######	######	######	######	######	######	######	######	######	######	######	######	######	792.26	######	######	######	######	######	######	######	######
Benzoic acid, ethyl ester	6.41	22.18	17.39	19.88	6.30	2.81	2.70	0.86	21.37	LOQ	LOQ	LOQ	LOQ	LOQ	LOQ	LOQ	0.95	0.94	0.48	0.79	LOD	LOD	LOD	LOD
Methyl anthranilate	162.79	230.68	149.31	229.24	136.13	132.71	105.33	102.99	42.22	1.03	1.03	1.02	0.91	1.01	1.01	0.71	14.60	16.81	8.50	7.94	LOD	LOD	LOD	LOD
Butanoic acid, phenylmethyl ester	######	######	######	######	915.10	######	913.98	######	4.12	1.02	1.02	1.02	0.92	0.99	0.99	0.69	29.64	34.95	34.31	59.07	226.41	180.04	138.88	155.82

3-Hexen-1-ol, benzoate, (Z)-	173.93	197.85	171.15	303.09	146.94	122.05	5.28	59.14	1.82	0.76	0.75	0.73	0.66	0.57	0.73	0.51	0.16	LOD	LOD	LOD	0.14	0.13	0.18	LOD
(Z, E)-alpha-Farnesene	LOD	LOD	LOD	LOD	0.24	0.10	0.23	0.28	6.83	0.51	0.49	1.09	0.98	0.84	0.55	0.39	517.27	721.29	445.54	638.69	######	616.93	480.46	398.13
	5.47	13.16	14.72	33.15	30.59	41.88	23.82	322.78	98.40	2.76	2.67	3.83	3.45	5.23	5.97	4.18	0.20	0.21	0.16	0.16	LOQ	LOQ	LOQ	LOQ
Benzyl alcohol	29.52	48.41	34.75	42.82	30.97	46.77	27.56	45.91	641.42	0.62	0.59	1.20	1.08	0.56	0.45	0.31	0.14	######	839.97	863.38	798.37	######	953.00	######
Linalool	192.43	340.65	280.48	450.90	250.02	285.46	181.04	285.50	0.11	3.48	4.05	7.87	7.08	6.38	5.08	3.56	0.32	0.21	0.14	0.11	0.21	0.36	0.26	0.30
Butanoic acid, ethyl ester	0.07	43.88	49.06	110.51	99.46	652.23	58.64	41.05	0.07	43.88	49.06	110.51	99.46	652.23	58.64	41.05	9.31	9.58	4.93	5.20	43.36	97.83	55.65	65.29
Acetic acid, butyl ester	LOD	LOD	LOD	LOD	LOD	LOD	LOD	LOD	0.42	4.43	4.04	6.23	5.61	5.64	3.85	2.69	30.29	15.99	8.50	53.04	LOQ	LOQ	LOQ	LOQ
2-Methylbut-2-en-1-yl acetate	LOD	LOD	LOD	LOD	LOD	LOD	LOD	LOD	0.43	0.92	1.24	1.01	0.91	1.14	1.16	0.81	0.59	0.65	0.31	0.40	0.40	0.43	0.25	LOD
3-Hexenoic acid, methyl ester	LOD	LOD	LOD	LOD	LOD	LOD	LOD	LOD	0.04	582.17	755.90	######	######	######	783.39	548.37	1.10	1.13	0.76	1.27	3.41	2.87	2.17	2.45
Butanoic acid, butyl ester	LOD	LOD	LOD	LOD	LOD	LOD	LOD	LOD	0.01	0.25	0.24	0.34	0.30	0.30	LOQ	LOQ	11.65	11.81	5.35	4.10	3.73	5.93	4.15	4.76
Acetic acid, hexyl ester	90.68	151.11	194.89	345.14	260.53	417.87	193.25	428.41	1.53	161.38	115.80	142.72	128.45	131.09	150.11	105.08	1.13	1.59	1.17	1.89	4.57	3.02	3.09	2.82
Pentanoic acid, 4-methyl-2-oxo-, methyl ester	0.01	0.14	0.04	0.19	0.03	0.32	0.06	1.09	0.04	0.18	0.17	0.25	0.23	0.24	0.26	0.19	3.04	3.61	1.99	2.36	0.12	0.12	0.12	5.53
Butanoic acid, 3-hexenyl ester, (Z)-	LOD	LOD	LOD	LOD	LOD	LOD	LOD	LOD	2.27	######	######	802.70	722.43	761.90	594.81	416.37	2.13	2.27	1.10	1.37	1.79	1.46	0.95	1.04
Methyl salicylate	52.38	106.47	135.51	142.03	90.85	98.84	70.06	88.04	174.61	0.14	LOD	0.18	0.16	0.15	LOD	LOD	2.09	2.15	1.18	1.18	3.43	1.17	0.90	1.02
cis-3-Hexenyl-alpha-methylbutyrate	3.77	6.83	7.93	15.65	9.85	4.52	3.02	6.27	12.55	4.00	4.20	10.00	9.00	11.58	9.81	6.87	52.55	50.31	20.26	19.06	13.86	23.49	14.29	15.17
Acetic acid, 2-phenylethyl ester	2.18	5.01	4.32	8.23	4.72	6.24	5.70	9.24	7.26	0.57	0.48	0.62	0.56	0.64	0.63	0.44	1.22	1.08	0.55	0.62	LOQ	LOQ	LOQ	LOQ
Benzoic acid, 2-hydroxy-, ethyl ester	LOD	1.23	1.77	3.53	4.51	1.86	1.36	3.12	0.91	169.49	140.46	308.20	277.38	387.22	385.61	269.93	######	######	742.69	743.77	758.74	######	904.58	739.37
n-Valeric acid cis-3-hexenyl ester	LOD	LOD	LOD	LOQ	LOQ	LOQ	LOD	LOD	0.93	######	######	######	######	2.55	3.18	2.22	55.74	80.11	78.44	72.84	151.42	145.15	117.35	89.47
2-Phenylethyl tiglate	LOQ	LOD	LOD	LOD	LOD	LOD	LOD	0.87	0.24	0.12	LOQ	0.29	0.26	1.11	0.96	0.67	0.14	LOQ	0.12	LOQ	0.12	0.12	0.12	0.12

Butyl benzoate	0.12	0.12	0.12	4.57	2.17	2.85	1.53	0.86	7.09	73.95	57.95	66.25	59.63	6.00	3.69	2.59	37.57	44.84	32.97	39.47	53.06	96.68	59.29	57.44
Hexanoic acid, 3-hexenyl ester, (Z)-	4.61	7.60	5.89	16.33	4.34	12.46	8.35	9.18	15.38	354.90	451.61	473.43	426.09	483.32	477.81	334.47	95.50	104.38	74.00	87.27	217.31	223.90	162.86	183.38
cis-3-Hexenyl cis-3-hexenoate	28.29	45.45	35.71	86.19	22.55	11.80	8.47	8.97	94.32	7.59	7.49	8.05	7.24	7.05	7.09	4.96	4.39	4.46	1.95	2.30	2.45	3.36	2.16	3.50
3-Hexen-1-ol, benzoate, (Z)-	180.41	337.92	293.25	312.97	121.36	207.09	161.30	98.19	8.27	0.96	1.02	0.95	0.85	0.96	0.96	0.68	0.12	0.21	0.14	0.41	0.75	1.56	1.55	1.81
beta-Myrcene	LOD	LOD	LOD	LOD	LOD	LOD	LOD	LOD	0.13	LOQ	0.16	0.38	0.34	0.59	0.56	0.39	LOQ	LOQ	LOQ	LOQ	0.24	0.18	0.18	0.16
D-Limonene	LOD	LOD	LOD	LOD	LOD	LOD	LOD	LOD	0.20	0.19	0.27	0.29	0.26	0.02	LOQ	LOQ	0.10	0.11	LOQ	LOQ	LOQ	LOQ	LOQ	LOQ
Bicyclo [3.1.0] hex-2-ene, 2-methyl-5-(1-methylethyl)-	LOD	LOD	LOD	5.96	5.06	3.67	2.40	9.16	0.50	6.67	5.20	8.85	7.97	12.58	12.37	8.66	0.29	0.48	0.35	0.40	4.74	2.30	1.19	1.25
3-Carene	LOD	LOD	LOD	LOD	2.47	LOD	LOD	4.50	2.69	LOQ	LOQ	LOQ	LOQ	LOD	LOD	LOD	65.06	61.91	24.39	22.50	15.10	21.58	13.08	13.26
alpha-Cubebene	LOQ	LOQ	LOQ	LOD	LOD	LOD	LOD	LOD	0.03	22.78	26.42	52.18	46.96	80.35	71.65	50.16	LOD	LOD	LOD	LOQ	0.21	LOD	LOD	LOQ
Copaene	LOD	LOD	LOD	LOD	LOD	LOD	LOD	LOD	7.38	16.69	14.40	27.45	24.70	21.52	17.37	12.16	7.20	8.30	4.75	6.75	11.43	13.16	10.35	11.97
Cyclohexane, 1-ethenyl-1-methyl-2,4-bis(1-methylethenyl)-,	10.81	11.39	12.36	11.74	LOD	LOD	LOD	LOD	0.04	5.54	2.36	8.35	7.52	22.38	17.73	12.41	2.34	3.32	2.29	3.44	5.40	4.87	4.07	3.82
1H-Cycloprop[e]azulene, 1a,2,3,4,4a,5,6,7b-octahydro	LOD	LOD	LOD	LOD	LOD	LOQ	LOQ	LOQ	0.23	4.10	5.89	11.78	10.60	7.78	9.87	6.91	LOQ	LOQ	LOQ	0.12	0.51	0.50	0.49	0.50
Caryophyllene	LOD	LOD	LOD	LOD	LOD	LOD	LOD	LOD	LOD	LOD	LOD	LOD	LOD	LOD	LOD	LOD	LOD	LOD	LOD	LOD	LOD	LOD	LOD	LOD
Aromandendrene	0.39	0.36	0.43	0.37	12.86	0.34	0.28	0.29	38.66	32.28	24.49	40.90	36.81	37.51	33.00	23.10	150.53	140.20	52.06	51.31	32.88	88.53	50.44	58.27
gamma-Muurolene	206.05	189.83	218.36	190.03	12.63	12.61	12.86	11.90	0.29	2.21	3.59	4.57	4.11	6.01	3.94	2.76	LOD	LOD	LOD	LOD	LOD	LOD	LOD	LOQ
alpha-Guaiene	LOD	LOD	LOD	LOD	LOD	LOD	LOD	LOD	1.40	29.41	25.13	35.17	31.65	32.23	23.75	16.63	0.11	0.14	LOD	LOD	0.22	0.21	0.15	0.19
α-Muurolene	LOD	LOD	LOQ	LOQ	LOQ	LOD	0.40	0.36	######	14.36	15.02	15.15	13.64	13.31	12.83	8.98	0.01	0.02	0.01	0.03	0.44	0.38	0.31	0.22
alpha-Farnesene	503.17	508.11	441.43	613.10	324.85	385.18	231.06	341.32	10.51	73.34	497.59	######	994.76	630.86	613.08	429.15	26.34	25.45	9.11	8.24	5.53	11.62	7.42	6.93
Naphthalene, 1,2,3,5,6,8a-hexahydro	3.15	2.95	2.88	3.30	3.52	3.33	3.66	3.61	0.51	0.33	0.31	0.30	0.27	0.30	LOD	LOD	1.80	2.23	1.17	1.25	1.40	1.86	1.23	0.87

Cubenene	2.04	1.94	1.89	2.12	2.29	2.19	2.39	2.39	41.13	LOQ	LOQ	LOQ	LOQ	LOQ	LOQ	LOQ	1.06	0.81	0.35	0.31	7.30	2.12	1.45	1.68
Naphthalene, 1,2,4a,5,6,8a-hexahydro-4,7	LOD	LOD	LOD	LOD	12.26	11.11	12.61	11.75	0.20	632.76	727.72	633.41	570.07	576.80	601.61	421.13	LOQ	LOQ	LOQ	LOQ	0.44	0.38	0.31	0.22
Bisabolol	LOD	LOD	LOD	LOD	LOD	LOD	LOD	LOD	4.64	25.35	19.63	54.43	48.99	51.40	42.15	29.50	1.29	1.82	0.94	1.05	1.19	2.02	1.28	1.49
1,6,10-Dodecatrien-3-ol, 3,7,11-trimethyl-, (E)-	######	169.86	146.93	260.18	126.16	104.79	81.24	50.79	0.00	1.46	1.94	1.28	1.15	1.44	1.27	0.89	LOD	LOD	LOQ	LOQ	LOQ	LOD	LOQ	LOQ
tau-Cadinol	LOD	LOD	LOD	LOQ	LOQ	LOQ	LOD	LOD	1.03	1.09	1.17	1.99	1.79	0.85	0.69	0.48	0.26	0.26	0.29	0.29	0.29	0.28	0.29	0.29
Benzene, 1-isocyano-3-methyl-	0.31	0.31	0.31	0.31	0.31	0.31	0.31	0.31	1.03	151.50	118.99	287.29	258.56	337.52	259.27	181.49	0.25	0.24	0.28	0.27	0.28	0.29	0.30	0.30
1H-Tetrazol-5-amine	0.31	0.31	0.31	0.30	0.31	0.31	0.31	0.30	18.49	2.13	2.46	9.75	8.77	4.62	2.91	2.03	6.81	7.04	5.19	4.46	4.52	9.52	7.91	7.29
indole	9.23	9.68	7.77	12.27	2.10	2.49	1.41	2.20	0.99	LOD	LOD	LOD	LOD	LOD	LOD	LOD	8.80	10.81	5.71	6.68	7.23	14.95	10.24	12.05
s-Triazolo[4,3-a] pyridine, 3,5,7-trimethyl-	0.31	0.31	0.31	0.31	0.31	0.31	0.31	0.31	0.25	0.27	0.33	0.27	0.24	0.21	0.20	0.14	0.31	0.31	0.31	0.31	0.26	0.27	0.28	0.26
3-Pyridinecarbonitrile, 1,4-dihydro-1-methyl-	LOD	0.15	0.17	0.13	0.17	0.16	0.17	0.14	79.20	37.07	34.15	38.35	34.51	42.94	40.21	28.15	6.81	7.04	5.19	4.46	4.52	9.52	7.91	7.29
Butanoic acid, 3-hexenyl ester, (E)-	23.76	50.85	42.15	92.46	33.62	14.89	11.15	29.16	0.23	######	######	######	######	92.13	8.23	5.76	3.72	5.38	3.93	6.34	8.97	13.40	11.74	12.66
5-Hexen-3-one	LOD	LOD	LOD	LOQ	LOQ	LOQ	LOD	0.11	1.12	0.39	0.56	0.57	0.51	0.57	0.58	0.40	0.58	0.62	0.43	0.46	0.80	1.08	1.03	LOD
Cyclohexene, 3-methyl-6-(1-methylethylidene)-	146.22	285.04	329.04	377.97	208.98	237.98	150.46	237.38	1.56	0.46	0.14	0.63	0.57	1.10	0.96	0.67	9.51	12.86	6.59	6.58	2.41	1.52	2.02	0.73
(E)-4,8-Dimethylnona-1,3,7-triene	0.47	0.66	1.08	1.37	1.03	0.80	0.39	0.70	1.71	2.46	2.23	5.11	4.60	7.63	6.81	4.77	0.43	0.57	0.36	0.62	1.53	LOD	LOQ	LOD
Cyclohexane, 1-ethenyl-1-methyl	0.51	0.44	0.58	0.38	0.44	0.43	0.43	0.39	12.38	8.77	7.93	14.31	12.88	16.87	16.55	11.59	7.57	8.45	5.54	7.06	9.63	14.96	11.51	13.70
3,7,11,11-Tetramethylbicyclo [8.1.0]-2,6-diene	3.71	8.82	7.54	10.55	5.12	11.29	4.23	5.90	12.38	6.94	7.42	7.34	6.61	7.04	7.52	5.26	1.43	1.75	1.18	1.53	1.87	2.98	2.38	2.22
methyl-3-methylenetric	3.71	4.31	4.51	4.55	4.06	4.39	3.88	3.87	7.68	5.09	4.72	8.20	7.38	9.35	9.28	6.50	LOD	LOQ	LOQ	LOQ	0.12	LOQ	LOQ	0.11

yclo
[4.4.0.02,7]
decane-rel-

Table S3 The qualitative and quantitative analysis of volatile components of the tea dhool

No.	Compounds	B. Identification Method			Linear Equations	R	Linear Range(μg/L)	LOD (μg/L)	LOQ (μg/L)
C19	Cis-3-Hexenyl Salicylate	MS	RI		Y=94.6014x-10.456	0.9923	1.995-100.000	0.0006	0.0389
C2	Epi-A-Cadinol	MS	RI		Y=38.0006x-0.3725	0.999	1.805-401.000	0.0009	0.0476
C2	Geraniol	MS	RI		Y=38.0006x-0.3725	0.999	1.805-401.000	0.0009	0.0476
C2	Methyl Anthranilate	MS	RI	Std	Y=38.0006x-0.3725	0.999	1.805-401.000	0.0009	0.0476
C20	(3z)-Hexenyl Butanoate	MS	RI	Std	Y=0.0717x+20.317	0.9983	2.001-20010.000	0.0362	0.0961
C20	Cis-3-Hexenyl Cis-3-Hexenoate	MS	RI		Y=0.0717x+20.317	0.9983	2.001-20010.000	0.0362	0.0961
C20	Cadinadiene	MS			Y=0.0717x+20.318	0.9983	2.001-20010.001	0.0362	0.0961
C20	Isoprppylcyclohexane	MS			Y=0.0717x+20.319	0.9983	2.001-20010.002	0.0362	0.0961
C21	Cis-3-Hexenyl Benzoate	MS	RI	Std	Y=15.3401x+0.1547	0.9625	2.013-10065.002	0.0319	0.0074
C21	A-Cadinene	MS			Y=15.3401x+0.1548	0.9625	2.013-10065.003	0.0319	0.0074
C21	Tetradecane	MS			Y=15.3401x+0.1549	0.9625	2.013-10065.004	0.0319	0.0074
C22	Cis-3-Hexenyl Isovalerate	MS	RI	Std	Y=0.002x+0.163	0.9993	2.024-202.400	0.0064	0.9436
C22	Hexanoic Acid, 3-Hexenyl Ester	MS	RI		Y=0.002x+0.164	0.9993	2.024-202.401	0.0064	0.9436
C22	3-Hexenyl Benzoate	MS	RI		Y=0.002x+0.165	0.9993	2.024-202.402	0.0064	0.9436
C23	Acetic Acid, Phenylmethyl Ester	MS	RI	Std	Y=0.3401x+0.1546	0.9921	2.042-25552.500	0.0050	0.5229
C24	Geranyl Acetate	MS	RI	Std	Y=10.5408x+0.6417	0.9983	2.011-2513.750	0.0137	1.1917
C24	(E)-3,7-Dimethyl-1,3,6-OctatRIene	MS			Y=10.5408x+0.6418	0.9983	2.011-2513.751	0.0137	1.1917
C24	Ocimene	MS	RI		Y=10.5408x+0.6419	0.9983	2.011-2513.752	0.0137	1.1917
C24	Ylangene	MS	RI		Y=10.5408x+0.6420	0.9983	2.011-2513.753	0.0137	1.1917
C25	(E)-3-Hexenyl Acetate	MS	RI	Std	Y=0.0655x+6.3373	0.9983	10.020-5010.000	0.0025	0.0019
C25	(Z)-3-Hexenyl Isobutanoate	MS	RI		Y=0.0655x+6.3374	0.9983	10.020-5010.001	0.0025	0.0019

C26	Indole	MS	RI	Std	Y=3.3042x+8.6759	0.9958	2.001-20010.000	0.0304	0.0186
C27	B-Myrcene	MS	RI	Std	Y=0.081x+0.930	0.9986	2.023-101.150	0.0026	0.0033
C27	A-Copaene	MS	RI		Y=0.081x+0.931	0.9986	2.023-101.151	0.0026	0.0033
C27	(-)-Beta-Elemene	MS	RI		Y=0.081x+0.932	0.9986	2.023-101.152	0.0026	0.0033
C27	Γ-Cadinene	MS	RI		Y=0.081x+0.933	0.9986	2.023-101.153	0.0026	0.0033
C27	Δ-Cadinene	MS	RI		Y=0.081x+0.934	0.9986	2.023-101.154	0.0026	0.0033
C28	(Z)-3-Hexenyl-2-Methylbutyrate	MS	RI	Std	Y=0.0003x-0.1845	0.9958	2.013-10065.000	0.0319	0.0032
C3	Benzyl Alcohol	MS	RI	Std	Y=10.0408x+36.417	0.9993	2.003-20030.001	0.1684	0.0044
C4	2-Methylnaphthalene	MS	RI	Std	Y=0.014x+18.96	0.995	2.033-20330.001	0.2889	0.1262
C5	D-Limonene	MS	RI	Std	Y=26.1214x+0.4572	0.9951	2.013-50.325	0.0010	0.0030
C5	Germacrene D	MS	RI		Y=26.1214x+0.4573	0.9951	2.013-50.326	0.0010	0.0030
C5	A-Zingiberene	MS	RI		Y=26.1214x+0.4574	0.9951	2.013-50.327	0.0010	0.0030
C5	A-Muurolene	MS	RI		Y=26.1214x+0.4575	0.9951	2.013-50.328	0.0010	0.0030
C5	A-Farnesene	MS	RI		Y=26.1214x+0.4576	0.9951	2.013-50.329	0.0010	0.0030
C5	1-Methylnaphthalene	MS			Y=26.1214x+0.4577	0.9951	2.013-50.330	0.0010	0.0030
C6	(Z)-3-Hexen-1-Ol	MS	RI	Std	Y=0.9029x-0.1055	0.9983	2.039-1274.375	0.0016	0.0996
C7	B-Cyclocitral	MS	RI	Std	Y=60.6010x+0.0181	0.9958	1.986-198.600	0.0010	0.0027
C8	B-Caryophyllene	MS	RI	Std	Y=3.3012x+0.1284	0.9911	2.014-2517.500	0.0363	0.0066
C8	Aromadendrene,	MS	RI		Y=3.3012x+0.1285	0.9911	2.014-2517.501	0.0363	0.0066
C8	Humulene	MS	RI		Y=3.3012x+0.1286	0.9911	2.014-2517.502	0.0363	0.0066
C8	(E)-B-Farnesene	MS	RI		Y=3.3012x+0.1287	0.9911	2.014-2517.503	0.0363	0.0066
C8	Γ-Muurolene	MS	RI		Y=3.3012x+0.1288	0.9911	2.014-2517.504	0.0363	0.0066
C8	B-Selinene	MS	RI		Y=3.3012x+0.1289	0.9911	2.014-2517.505	0.0363	0.0066
C9	Benzaldehyde	MS	RI	Std	Y=1.7023x-0.210	0.9954	2.032-2540.000	0.0022	0.1063
C1	(E)-Nerolidol	MS	RI	Std	Y=0.0686x+78.41	0.9958	2.013-10065.001	0.0319	0.8736
C10	Methyl Benzoate	MS	RI	Std	Y=5.2054x-3.3047	0.9568	2.035-40700.000	0.3249	0.0276

C11	Acetic Acid, 2-Phenylethyl Ester	MS	RI		Y=0.0003x+0.0624	0.9911	2.011-5027.501	0.0127	0.0145
C11	Benzoic Acid, Ethyl Ester	MS	RI	Std	Y=0.0003x+0.0624	0.9911	2.011-5027.501	0.0127	0.0145
C11	Butyl Benzoate	MS	RI		Y=0.0003x+0.0624	0.9911	2.011-5027.501	0.0127	0.0145
C11	Benzyl Benzoate	MS	RI		Y=0.0003x+0.0624	0.9911	2.011-5027.501	0.0127	0.0145
C12	N-Hexyl Benzoate	MS	RI	Std	Y=0.4553x+0.0773	0.9994	10.000-10098.000	0.0461	0.1392
C13	Phenylethyl Alcohol	MS	RI	Std	Y=13.709x-18.389	0.9923	1.996-79920.000	0.3923	0.0067
C13	Benzene Acetonitrile	MS	RI		Y=13.709x-18.389	0.9923	1.996-79920.000	0.3923	0.0067
C14	Butanoic Acid, Phenylmethyl Ester	MS	RI	Std	Y=2.8403x-0.1845	0.994	2.011-402.200	0.0011	0.0147
C15	Eugenol	MS	RI	Std	Y=13.409x-0.2455	0.9951	2.004-400.800	0.0030	0.0940
C15	(1r)-1α-Isopropyl-6-Octahydronaphthalene	MS			Y=13.409x-0.2455	0.9951	2.004-400.800	0.0030	0.0940
C15	1-Methoxy-4-Benzene	MS			Y=13.409x-0.2455	0.9951	2.004-400.800	0.0030	0.0940
C16	Linalool Oxide I	MS	RI		Y=0.4311x-0.1321	0.9981	10.000-100098.000	0.0471	0.1421
C16	Linalool	MS	RI	Std	Y=0.4311x-0.1321	0.9981	10.000-100098.000	0.0471	0.1421
C16	Cubebene	MS			Y=0.4311x-0.1321	0.9981	10.000-100098.000	0.0471	0.1421
C17	Linalool Oxide Ii	MS	RI		Y=3.5019x+0.1465	0.9986	10.000-100098.001	0.0471	0.0089
C17		MS	RI	Std	Y=3.5019x+0.1465	0.9986	10.000-100098.001	0.0471	0.0089
C17	Cis-Muurola-4(14),5-Diene	MS			Y=3.5019x+0.1465	0.9986	10.000-100098.001	0.0471	0.0089
C18	Methyl Salicylate	MS	RI	Std	Y=3.9123x-0.0254	0.995	1.999-199.900	0.0083	0.1421
C18	Epi-Bicyclosesquiphellandrene	MS			Y=3.9123x-0.0254	0.995	1.999-199.900	0.0083	0.1421
C19	Benzoic Acid, 2-Hydroxy-, Ethyl Ester	MS	RI	Std	Y=94.6014x-10.456	0.9923	1.995-100.000	0.0006	0.0389

Note. b. Method of identification: MS, Comparison of mass spectra with MS libraries and retention times. RI, retention index. Std, confirmed by authntic standards. LOD: Limit of detection. LOQ: Limit of quantitation.

Table S4 The quantitative results of volatile constituents of jasmine tea

Compounds	The quantitative of volatile components of the new scenting process jasmine flower									The quantitative of volatile components of the traditional scenting process tea base								
	0.00	2.00	4.00	6.00	8.00	10.00	12.00	14.00	Finished	0.00	2.00	4.00	6.00	8.00	10.00	12.00	14.00	Finished

(-)-beta-Elemene	35.70	57.59	32.68	46.28	83.30	36.14	57.76	46.16	21.69	56.31	12.38	20.77	23.54	42.04	42.88	62.25	36.07	39.44
(1R)-1α-octahydronaphthalene	LOD	0.16	0.05	0.12	0.21	LOD	0.14	LOD	0.10	0.11	LOD	LOD	LOD	LOD	0.12	0.15	0.11	0.14
(3Z)-Hexenyl butanoate	618.86	201.67	556.65	455.89	820.61	582.48	728.11	608.61	552.60	327.86	351.82	177.37	196.32	301.61	190.40	288.79	231.06	262.72
(E)-3,7-Dimethyl-1,3,6-octatriene	0.23	0.53	0.36	1.03	1.85	1.06	0.59	0.83	0.59	0.42	LOD	LOQ	1.34	1.68	0.80	1.50	0.25	1.54
(E)-3-Hexenyl acetate	452.88	254.78	352.41	843.46	1518.22	1104.09	863.01	1375.28	1224.91	402.56	297.87	422.46	693.52	1177.87	1323.43	2038.55	1203.25	1832.35
(E)-Nerolidol	LOD	3.70	1.03	2.30	4.14	0.44	12.04	3.40	5.60	0.73	0.64	1.72	1.24	1.45	6.31	2.77	2.69	5.70
(E)-β-Farnesene	0.68	1.16	0.63	1.00	1.80	LOD	1.14	0.98	0.61	0.95	0.47	0.71	0.71	0.88	0.83	1.22	0.84	0.64
(Z)-3-Hexen-1-ol	13.59	33.88	14.19	78.42	141.16	86.46	77.86	146.00	121.00	15.97	LOD	47.92	89.60	124.54	218.75	346.82	233.23	306.30
(Z)-3-Hexenyl isobutanoate	0.15	2.61	LOD	LOD	LOD	LOQ	1.09	0.36	0.06	LOD	LOD	LOD	LOD	LOD	LOQ	LOQ	LOQ	0.57
(Z)-3-hexenyl-2-methylbutyrate	1.39	1.21	1.25	1.30	2.34	1.62	3.51	2.23	2.12	1.02	0.80	0.90	0.74	1.20	1.36	1.85	1.46	1.64
1-Methoxy-4-(1-propenyl)-benzene	0.19	0.76	0.46	1.10	1.98	0.74	0.29	0.79	0.12	0.79	0.53	0.38	0.22	0.19	0.37	0.82	0.68	0.07
1-methylnaphthalene	LOD	0.13	0.08	0.12	0.22	0.20	LOD	0.10	0.01	LOD	LOD	LOQ	LOQ	0.15	0.14	0.18	LOD	0.05
2-methylnaphthalene	LOD	LOD	LOD	LOD	LOD	LOD	LOQ	LOQ	0.05	LOD	LOD	LOD	LOD	LOQ	LOD	LOQ	LOQ	0.04
3-Hexenyl benzoate	0.48	1.83	0.75	1.38	2.48	0.81	1.96	1.68	1.75	0.87	0.78	1.07	0.99	1.21	1.70	1.86	1.65	1.90
Acetic acid, 2-phenylethyl ester	0.11	0.23	0.09	0.18	0.33	0.13	0.16	0.17	1.02	0.71	0.57	0.96	1.05	1.35	1.19	2.12	1.40	1.92
Acetic acid, phenylmethyl ester	717.17	1364.96	1003.33	1855.42	3339.76	1199.76	967.47	1505.63	1139.06	1267.50	965.34	1223.91	899.31	1053.67	984.82	1398.95	1107.71	1124.57
Aromadendrene,	LOD	0.35	0.15	0.34	0.62	0.31	LOQ	0.29	0.22	0.28	0.17	0.18	LOD	0.28	0.25	0.39	0.23	0.08
bate-Copaene	1.15	2.10	1.07	0.33	0.60	0.18	0.32	0.30	0.29	0.20	0.20	0.17	0.23	1.56	1.61	2.28	1.41	0.37
Benzaldehyde	1.02	2.34	0.75	2.27	4.09	0.32	0.65	0.31	1.68	2.92	1.56	1.52	0.88	1.09	0.29	1.85	1.79	2.28
benzene acetonitrile	397.92	574.67	431.89	659.04	1186.27	553.46	406.73	610.58	497.69	511.28	391.09	459.94	371.83	434.64	464.48	549.84	294.89	449.16
Benzoic acid, 2-(methylamino)-, methyl ester	0.28	1.42	0.43	1.09	1.96	0.63	0.84	1.32	1.09	0.64	0.41	1.17	1.08	1.39	2.42	2.73	2.71	3.02
Benzoic acid, ethyl ester	LOD	0.06	0.53	0.29	0.53	0.18	0.81	1.01	0.94	LOD	1.05	LOD	LOD	0.36	1.15	1.47	1.83	0.90
Benzoic acid, ethyl ester	0.77	3.69	1.20	5.90	10.63	3.13	4.41	4.20	3.93	1.43	0.34	4.86	4.09	4.58	4.05	6.10	5.89	8.19
benzyl alcohol	1330.62	3714.29	934.66	3476.50	6257.70	2510.52	2343.03	2951.77	33.05	2773.99	1563.72	3976.79	3891.19	4156.98	6064.26	9982.25	6511.68	34.80
Benzyl benzoate	LOQ	LOD	0.26	LOD	LOD	0.30	1.29	0.48	0.83	LOD	0.87	2.15	0.29	0.63	1.09	2.82	2.40	0.73
Butanoic acid, phenylmethyl ester	0.48	1.80	1.18	1.89	3.39	1.07	2.49	1.86	1.56	0.65	0.85	1.47	0.95	1.30	1.44	1.70	1.91	1.55
Butyl benzoate	LOD	0.39	0.17	0.30	0.54	0.19	0.54	0.34	0.39	0.31	0.18	0.35	0.28	0.32	0.39	0.46	0.37	0.40

cis-3-Hexenyl benzoate	22.24	100.63	40.82	74.67	134.40	40.31	100.72	82.37	80.34	47.33	42.94	59.46	51.22	61.93	80.60	84.16	72.56	86.17
Cis-3-Hexenyl cis-3-hexenoate	213.31	107.01	271.59	217.78	392.01	209.03	392.72	284.56	203.31	105.46	196.67	82.32	99.44	134.35	82.87	114.78	96.63	113.68
cis-3-Hexenyl isovalerate	10.64	11.38	18.46	14.97	26.95	17.11	100.82	37.73	69.48	8.36	9.52	10.66	13.12	11.10	46.11	65.14	54.82	55.85
cis-3-Hexenyl salicylate	LOD	LOD	LOD	LOD	LOD	LOD	LOD	LOD	0.01	0.51	LOD	LOD	LOD	LOD	LOQ	LOD	LOD	0.01
cis-Muurola-4(14),5-diene	2.41	4.45	1.40	3.26	5.87	2.58	3.79	3.36	3.57	2.59	1.32	0.63	2.65	3.49	3.57	5.51	1.97	5.07
Cubebene	LOD	LOD	LOD	LOD	0.00	LOD	LOQ	LOQ	0.00	LOD	LOD	LOQ	LOD	LOQ	LOD	0.11	LOD	0.08
D-Limonene	0.23	0.19	0.15	0.37	0.67	0.29	0.19	0.25	0.22	0.28	LOD	LOQ	0.47	0.50	0.27	0.45	0.18	0.44
bicyclosesquiphellandrene	1.37	4.64	1.67	4.94	8.89	2.10	6.08	3.50	2.63	3.09	1.64	1.29	2.11	3.24	4.30	7.51	17.25	4.73
epi-α-Cadinol (syn. τ-cadinol)	LOQ	11.06	0.35	1.98	3.57	2.13	4.54	2.16	2.80	4.23	0.58	0.33	0.82	2.04	5.52	3.88	5.06	5.00
Eugenol	1.57	2.36	1.41	2.78	5.00	3.08	2.55	2.53	2.84	1.07	0.94	2.15	3.93	3.95	10.74	5.45	7.67	9.40
geraniol	0.35	1.08	0.55	2.28	4.10	1.33	1.28	1.25	0.33	0.13	0.14	0.11	LOD	0.11	0.30	0.25	0.34	0.36
Geranyl acetate	2.24	1.77	2.36	2.81	5.06	1.70	13.18	4.44	8.94	1.63	2.53	2.68	2.57	5.02	7.60	9.37	7.65	9.13
Germacrene D	4.10	9.73	3.11	4.15	7.48	2.20	8.06	4.35	1.59	4.71	2.03	2.94	LOQ	1.98	4.15	6.71	5.02	3.52
Hexanoic acid, 3-hexenyl ester	35.08	13.10	35.95	22.56	40.61	25.05	44.05	30.98	23.89	13.38	21.73	7.31	6.12	9.28	5.55	9.26	7.81	8.91
Humulene	33.88	52.72	27.00	27.17	48.90	23.20	42.99	27.01	14.08	49.04	19.60	25.41	16.01	26.13	28.34	42.11	25.66	22.80
Indole	56.01	254.91	85.35	221.48	398.66	122.97	181.31	199.68	178.04	148.86	100.67	206.24	158.31	180.66	221.96	262.95	240.79	210.50
isoprppylcyclohexane	34.26	82.77	57.26	92.87	167.16	81.48	203.94	146.21	126.65	57.78	38.67	72.69	57.77	93.14	140.97	188.42	151.69	153.12
linalool	200.06	551.21	271.19	625.02	1125.04	405.11	438.15	524.73	416.14	524.36	227.09	489.32	319.71	403.42	477.99	586.18	467.77	492.65
linalool oxide I	0.21	0.12	0.10	0.34	0.61	0.27	0.12	0.35	0.51	0.14	LOD	LOQ	0.12	0.21	0.17	0.13	0.17	0.26
linalool oxide II	0.69	1.56	0.55	1.53	2.75	1.25	0.99	1.87	1.52	1.15	0.38	1.28	0.92	1.25	2.05	2.99	2.53	2.79
Methyl anthranilate	21.33	102.05	28.19	86.15	155.07	45.33	80.24	79.40	78.46	54.91	39.35	93.51	75.11	89.12	128.40	144.90	138.74	127.12
Methyl benzoate	83.23	503.94	150.28	419.07	754.33	246.11	194.85	280.18	219.43	407.71	124.61	447.73	248.16	281.75	24.41	291.46	201.73	217.90
methyl salicylate	26.96	131.16	45.78	128.30	230.94	78.14	97.57	130.53	160.51	98.96	38.15	126.53	84.83	111.17	152.91	194.63	149.96	100.55
n-Hexyl benzoate	0.97	3.78	1.69	2.47	4.44	1.38	3.44	3.43	2.59	2.27	2.01	2.49	1.71	2.22	2.70	3.52	2.90	2.85
Ocimene	0.69	0.26	0.46	1.21	2.17	1.33	1.18	0.91	1.24	0.68	0.29	0.42	1.59	2.18	1.96	3.53	1.36	3.02
phenylethyl alcohol	1681.27	1071.09	887.78	1198.94	2158.09	1539.44	569.82	986.16	963.19	1156.49	758.48	1077.02	591.69	697.82	780.87	1113.87	972.05	672.25
tetradecane	53.51	41.45	32.58	48.19	86.75	47.64	30.98	20.32	LOD	39.92	24.48	36.61	20.54	29.74	23.98	33.43	34.90	LOD
Ylangene	0.32	0.70	0.28	0.64	1.16	0.40	0.62	0.51	0.43	0.47	0.30	0.22	0.37	0.59	0.70	0.79	0.51	0.90
α-cadinene	0.21	0.56	0.22	0.44	0.79	0.30	0.22	0.45	0.35	0.39	0.29	0.24	0.32	0.42	0.41	0.58	0.36	0.40

α-Copaene	1.90	5.30	0.38	3.32	5.97	1.41	3.48	2.19	0.51	3.61	LOD	0.53	0.83	2.84	2.51	4.36	1.93	0.77
α-farnesene	318.50	608.97	303.07	453.18	815.72	318.99	534.42	514.10	291.24	485.98	274.80	416.41	276.81	429.57	426.72	643.97	442.93	293.65
α-Muurolene	0.40	0.74	0.35	0.65	1.16	0.40	0.82	0.57	0.49	0.47	0.33	0.37	0.44	0.53	0.70	0.94	0.57	0.66
α-zingiberene	198.61	366.76	193.68	318.32	572.97	245.66	394.87	334.95	239.11	300.61	168.00	211.03	272.13	345.91	296.93	483.58	279.98	270.37
β-caryophyllene	6.34	8.33	2.53	2.97	5.34	2.37	8.79	2.38	0.70	9.15	0.23	2.82	LOD	6.41	6.04	5.99	5.92	1.20
β-cyclocitral	6.77	7.29	4.97	2.44	4.39	3.55	LOD	1.11	1.71	5.30	4.12	0.95	LOQ	LOQ	LOD	LOD	LOD	0.08
β-myrcene	0.57	0.55	0.27	0.43	0.77	2.08	1.16	1.31	2.06	1.20	0.26	0.23	4.85	4.79	1.82	3.94	0.65	4.35
β-selinene	1.33	2.90	1.28	1.85	3.33	1.63	0.76	1.55	1.69	1.42	0.69	0.77	LOD	0.14	0.38	0.42	0.54	0.73
γ-Cadinene	1.20	3.39	1.44	2.47	4.45	1.54	0.80	2.62	1.93	2.23	1.74	1.55	1.65	2.20	2.22	2.99	2.12	2.25
γ-Muurolene	41.19	60.71	32.89	22.69	40.84	17.87	45.85	29.04	14.14	53.34	27.23	35.25	8.11	15.16	22.52	28.03	26.13	11.91
δ-Cadinene	1.80	3.32	1.66	2.49	4.48	1.74	3.30	2.49	1.93	2.23	1.64	1.55	1.65	2.28	2.41	3.33	2.16	2.59

Table S5 The variations of OAV values of aroma components in jasmine flower and jasmine tea

Compounds	Oders description	Oders attributes	Threshold (μg/L)	Odor activity value (OAV) of the traditional scenting process jasmine								Odor activity value (OAV) of the new scenting process jasmine								Odor activity value (OAV) of the jasmine flower								
				0h	2h	4h	6h	8h	10h	12h	14h	0h	2h	4h	6h	8h	10h	12h	14h	0h	2h	4h	6h	8h	10h	12h	14h	Finished
Phytol acetate	Banana fragrance	Floral	750	7.82	9.70	12.60	22.38	17.00	27.29	12.56	28.03	19.38	0.55	0.53	0.59	0.53	0.61	0.60	0.42	0.30	0.30	0.15	0.21	0.22	0.21	0.14	0.15	0.10
Methyl benzoate	Similar to pear and apricot aroma	Fragrant	110	12.80	30.77	29.20	21.89	8.39	11.71	5.33	5.97	42.67	0.91	0.84	1.38	1.25	1.38	1.37	0.96	0.10	0.11	0.05	0.07	-	-	-	-	0.03
Benzyl acetate	Jasmine floral, fruity	Floral	270	56.54	102.49	74.25	110.77	49.87	74.98	49.50	63.81	183.62	42.06	48.38	82.14	73.92	53.50	41.92	29.34	72.70	75.37	47.40	48.26	78.72	70.35	51.18	46.44	3.22
Ethyl benzoate	Fruity	Fragrant	30	8.02	27.73	21.74	24.85	7.88	3.51	3.38	1.07	26.72	0.01	0.01	0.01	0.01	0.01	0.01	0.01	1.18	1.17	0.60	0.98	-	-	-	-	-
Methyl 2-aminobenzoate	Grape fragrance	Floral	3	542.65	768.93	497.69	764.13	453.77	442.36	351.11	343.31	140.72	3.42	3.42	3.38	3.05	3.36	3.36	2.35	48.65	56.02	28.33	26.48	-	-	-	-	-
Benzyl butyrate	Similar to pear and apricot aroma	Fragrant	200	103.01	187.58	135.82	202.74	91.51	137.84	91.40	118.06	0.41	0.10	0.10	0.10	0.09	0.10	0.10	0.07	2.96	3.49	3.43	5.91	22.64	18.00	13.89	15.58	2.48
N-hexyl benzoate	Apple aroma	Fragrant	30	57.98	65.95	57.05	101.03	48.98	40.68	1.76	19.71	0.61	0.25	0.25	0.24	0.22	0.19	0.24	0.17	0.05	-	-	-	0.05	0.04	0.06	-	0.13

alpha-farnesene	Cooked vegetables, flowers, wood	Fragrant	450	33.54	33.87	29.43	40.87	21.66	25.68	15.40	22.75	0.70	4.89	33.17	73.69	66.32	42.06	40.87	28.61	1.76	1.70	0.61	0.55	0.37	0.77	0.49	0.46	0.01
Cis-3-hexenol	Green tea fragrance	Fragrant	70	0.78	1.88	2.10	4.74	4.37	5.98	3.40	46.11	14.06	0.39	0.38	0.55	0.49	0.75	0.85	0.60	0.03	0.03	0.02	0.02	-	-	-	-	0.10
Benzyl alcohol	Green smell	Fragrant	400	7.38	12.10	8.69	10.70	7.74	11.69	6.89	11.48	160.36	0.16	0.15	0.30	0.27	0.14	0.11	0.08	0.03	497.00	209.99	215.84	199.59	390.33	238.25	304.63	111.19
Linalool	Sweet floral	Floral	10	192.43	340.65	280.48	450.90	250.02	285.46	181.04	285.50	0.11	3.48	4.05	7.87	7.08	6.38	5.08	3.56	0.32	0.21	0.14	0.11	0.21	0.36	0.26	0.30	0.13

Compound,	Oders description	Oders attributes	Threshold (μg/L)	Odor activity value (OAV) of the new scenting process tea									Odor activity value (OAV) of the traditional scenting process tea								
				0h	2h	4h	6h	8h	10h	12h	14h	Finished	0h	2h	4h	6h	8h	10h	12h	14h	Finished
Phytol acetate	Banana fragrance	Floral	750	6.04	3.40	4.70	11.25	12.37	14.72	11.51	18.34	16.33	3.97	5.63	9.25	15.70	17.65	27.18	16.04	24.43	2.44
Methyl benzoate	Similar to pear and apricot aroma	Fragrant	110	7.57	45.81	13.66	38.10	41.91	22.37	17.71	25.47	19.95	11.33	40.70	22.56	25.61	2.22	26.50	18.34	19.81	1.98
Benzyl acetate	Jasmine floral, fruity	Floral	270	26.56	50.55	37.16	68.72	75.59	44.44	35.83	55.76	42.19	35.75	45.33	33.31	39.02	36.47	51.81	41.03	41.65	4.17
Ethyl benzoate	Fruity	Fragrant	30	-	0.02	0.18	0.10	0.11	0.06	0.27	0.34	0.31	-	-	0.12	0.35	0.38	0.49	0.61	0.30	0.03
Methyl 2-aminobenzoate	Grape fragrance	Floral	3	71.10	340.16	93.97	287.16	315.88	151.08	267.47	264.66	261.55	131.16	311.71	250.36	297.06	428.00	482.98	462.47	423.74	42.37
Benzyl butyrate	Similar to pear and apricot aroma	Fragrant	200	-	0.01	0.01	0.02	0.02	0.02	0.06	0.02	0.04	0.04	0.11	0.01	0.03	0.05	0.14	0.12	0.04	0.00
N-hexyl benzoate	Apple aroma	Fragrant	30	0.32	1.26	0.56	0.82	0.90	0.46	1.15	1.14	0.86	0.67	0.83	0.57	0.74	0.90	1.17	0.97	0.95	0.09
alpha-farnesene	Cooked vegetables, flowers, wood	Fragrant	450	7.08	13.53	6.73	10.07	11.08	7.09	11.88	11.42	6.47	6.11	9.25	6.15	9.55	9.48	14.31	9.84	6.53	0.65
Cis-3-hexenol	Green tea fragrance	Fragrant	70	1.94	4.84	2.03	11.20	12.32	12.35	11.12	20.86	17.29	-	6.85	12.80	17.79	31.25	49.55	33.32	43.76	4.38

Benzyl alcohol	Green smell	Fragrant	400	0.19	0.92	0.30	1.48	1.62	0.78	1.10	1.05	0.98	0.09	1.22	1.02	1.15	1.01	1.53	1.47	2.05	0.02
Linalool	Sweet floral	Floral	10	200.06	551.21	271.19	625.02	687.52	405.11	438.15	524.73	416.14	227.09	489.32	319.71	403.42	477.99	586.18	467.77	492.65	49.27

Note: '-' indicates that OAV can't be calculated

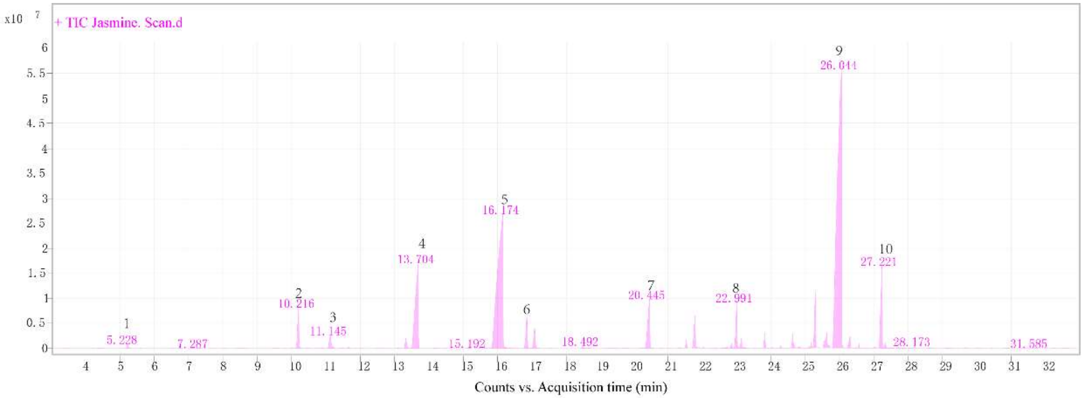

Figure S1. The main identified compounds of jasmine in TIC.

Note: 1, (Z)-3-Hexen-1-ol; 2, Acetic acid, hexyl ester; 3, Benzyl alcohol; 4, Linalool; 5, Acetic acid, phenylmethyl ester; 6, Benzoic acid, ethyl ester; 7, Indole; 8, Butyl benzoate; 9, α-Farnesene; 10. (Z)-3-Hexen-1-ol, benzoate.

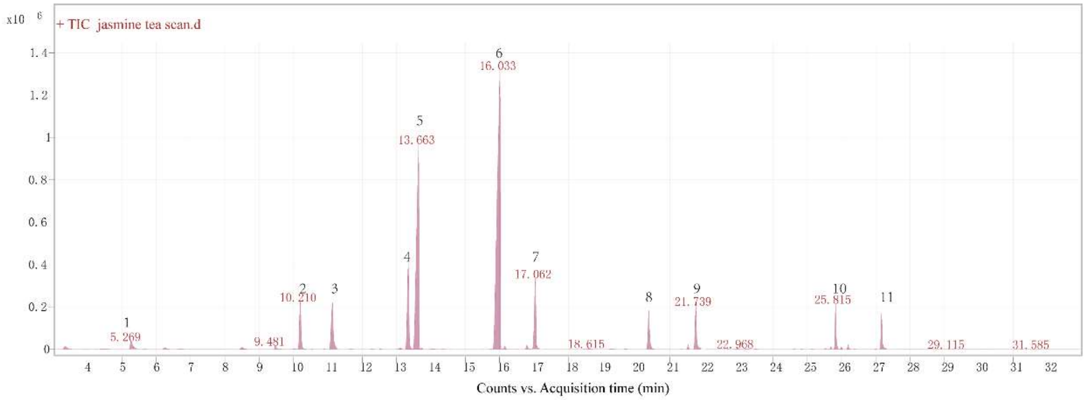

Figure S2. The main identified compounds of jasmine tea in TIC.

Note: 1, (Z)-3-Hexen-1-ol; 2, (E)-3-Hexenyl acetate; 3, Benzyl alcohol; 4, Methyl benzoate; 5, Linalool; 6, Benzoic acid, ethyl ester; 7, Methyl salicylate; 8, Indole; 9, Methyl anthranilate; 10, α-Farnesene; 11, cis-3-Hexenyl benzoate.
